# Epstein-Barr Virus-Induced Genes and Endogenous Retroviruses in Immortalized B Cells from Patients with Multiple Sclerosis

**DOI:** 10.3390/cells11223619

**Published:** 2022-11-15

**Authors:** Lisa Wieland, Tommy Schwarz, Kristina Engel, Ines Volkmer, Anna Krüger, Alexander Tarabuko, Jutta Junghans, Malte E. Kornhuber, Frank Hoffmann, Martin S. Staege, Alexander Emmer

**Affiliations:** 1 Department of Neurology, Medical Faculty, Martin Luther University Halle-Wittenberg, 06120 Halle (Saale), Germany; 2 Department of Surgical and Conservative Pediatrics and Adolescent Medicine, Medical Faculty, Martin Luther University Halle-Wittenberg, 06120 Halle (Saale), Germany; 3 Department of Neurology, Martha-Maria Hospital Halle-Dölau, 06120 Halle (Saale), Germany

**Keywords:** multiple sclerosis, Epstein-Barr virus, lymphoblastoid cell lines, human endogenous retrovirus, biomarkers

## Abstract

The immune pathogenesis of multiple sclerosis (MS) is thought to be triggered by environmental factors in individuals with an unfavorable genetic predisposition. Epstein–Barr virus (EBV) infection is a major risk factor for subsequent development of MS. Human endogenous retroviruses (HERVs) can be activated by EBV, and might be a missing link between an initial EBV infection and the later onset of MS. In this study, we investigated differential gene expression patterns in EBV-immortalized lymphoblastoid B cell lines (LCL) from MS-affected individuals (MSLCL) and controls by using RNAseq and qRT-PCR. RNAseq data from LCL mapped to the human genome and a virtual virus metagenome were used to identify possible biomarkers for MS or disease-relevant risk factors, e.g., the relapse rate. We observed that lytic EBNA-1 transcripts seemed to be negatively correlated with age leading to an increased expression in LCL from younger PBMC donors. Further, HERV-K (HML-2) GAG was increased upon EBV-triggered immortalization. Besides the well-known transactivation of HERV-K18, our results suggest that another six HERV loci are up-regulated upon stimulation with EBV. We identified differentially expressed genes in MSLCL, e.g., several HERV-K loci, ERVMER61-1 and ERV3-1, as well as genes associated with relapses. In summary, EBV induces genes and HERV in LCL that might be suitable as biomarkers for MS or the relapse risk.

## 1. Introduction

The Epstein–Barr virus (EBV) is the causative agent of infectious mononucleosis (IM), also known as Pfeiffer’s glandular fever. After infection, the virus stays present latently in the B cell memory pool and persists in the human body over a lifetime, without triggering symptoms [1,2]. If the immune system is weakened, the virus-infected B cells can proliferate uncontrollably, and EBV can become a trigger for lymphoproliferative disorders [3,4]. Besides cancer, EBV infection is thought to be a major risk factor for the development of multiple sclerosis (MS), since seropositivity to EBV nuclear antigen (EBNA) epitopes or the viral capsid antigen (VCA) is more prevalent among MS-affected individuals than in controls [5,6,7,8]. A very recent report from MS cases among US military personnel suggests a 32-fold increased risk of MS and higher serum levels of neurofilament light chain, a biomarker of neuroaxonal degeneration, after EBV seroconversion [9]. Furthermore, similarities between the epidemiology of MS and a delayed EBV primary infection manifesting as IM were observed, including the latitude gradient and earlier onset in women than in men [10,11]. As a result, an IM diagnosis in adolescents and young adults has been associated with a two- to fourfold increased risk of MS [12,13,14,15]. However, exposure to EBV alone is not sufficient to develop MS. A combination of environmental and genetic factors appears to be involved, resulting in the dysregulation of the immune system [16,17]. In the last decades, human endogenous retroviruses (HERVs) have increasingly been associated with nervous system disorders, e.g., based on the increased expression of different HERV loci in various cell types, tissues, and biological fluids from individuals with MS (reviewed in [18]). Further, the appearance of retrovirus-like particles in B cells from the cerebrospinal fluid (CSF) of an individual with MS supported the hypothesis of a putative role in the pathogenesis of MS [19]. HERVs represent stably integrated copies of exogenous viruses in the human genome that have been “endogenized” during evolution and are commonly inactivated [20]. Nevertheless, some HERVs still have relatively intact open reading frames, e.g., for envelope proteins, which can have immunostimulatory and pro-inflammatory properties like superantigens [21,22,23]. HERVs can be transactivated by EBV [24,25], suggesting that there may be a connection between EBV, HERVs and MS.

In this paper we aimed to study the influence of an EBV-driven proliferation on the gene expression in B cells from individuals with MS. We used the fact that EBV can immortalize B cells in vitro and generate so-called lymphoblastoid cell lines (LCL) [26,27]. Concerning B cells that have been observed in MS brain lesions of early and progressive phases of MS [28] and the development of successful B cell-depleting therapies in the relapsing-remitting and primary progressive forms of MS [29], these LCL might overcome some limitations of traditional murine models of MS that are mediated largely by pathogenic T cells. In this study, we wanted to investigate putative differential expression patterns in MS and controls. Therefore, multiple LCL from 32 individuals with MS and 10 controls were established and analyzed by RNAseq. As HERVs, and especially their protein coding regions (e.g., ENVs), are discussed in the pathogenesis of MS, we secondly aimed to investigate for differentially expressed HERV within our LCLs from MS and controls. We used a synthetic virus metagenome designed by our group to simultaneously compare the expression of more than a hundred HERVs, providing the most complete open-reading frames for their coding regions or even nearly complete proviral sequences by RNAseq analyses. Further, we focused on possible correlations of environmental and clinicopathological features with the activation of the EBV lytic and latent cycle, and selected HERV transcripts using RNAseq and quantitative reverse transcription-polymerase chain reaction (qRT-PCR). In this regard, we focused on two HERV-families, namely HERV-W and HERV-K, as they are frequently discussed to play a role in the pathogenesis of MS (reviewed in [30,31,32]). For the HERV-W family, we examined the envelope from ERVWE-1 on chromosome 7 (HERV-W1 *ENV*), and another envelope with a yet unknown genomic location named MS-associated retrovirus (MSRV *ENV*). For HERV-K, the group specific antigen (GAG) region was chosen as the target region, because it allows the detection of numerous members of the most biologically active HERV family without distinguishing between type 1 and type 2 HERV-K proviruses. In qRT-PCR analyses, we took advantage of this fact and used specific primers to detect multiple members of the HERV-K (HML-2) family based on their high sequence homology. Since this was not possible for RNAseq analyses, we investigated the expression of a HERV, which is also targeted by qRT-PCR, namely HERV-K4. To monitor the activation of EBV, we detected the major transcript expressed in LCL during latent cycle progression, called EBNA-2, as well as EBNA-1 starting at the F promoter site, which is used in the lytic EBV cycle [33,34,35,36].

## 2. Materials and Methods

### 2.1. Cell Lines and Cell Culture 

Peripheral blood mononuclear cells (PBMCs) were isolated by density gradient centrifugation [37] from 10 controls and from 32 individuals with MS with informed consent and with approval by the ethics committee of the Martin Luther University Halle-Wittenberg (protocol code: 2015-89). Within our study cohort the mean age was 39.1 (range: 23–56), and approximately two-thirds (n = 21) were female. All individuals with MS had a diagnosed relapsing–remitting course of MS when they joined the study. The mean duration of the disease was 7.3 years (range 0–21 years). The mean EDSS score was 2.9 (range: 1–5.5). For one individual with MS no EDSS assessment was possible. Of all the individuals with MS, 22 received therapies including treatment with natalizumab (n = 14), fingolimod (n = 3), alemtuzumab (n = 1), interferon beta-1a (n = 1), carbamazepine (n = 1), glatiramer acetate (n = 1) or dimethyl fumarate (n = 1) at the time of blood collection. Ten individuals with MS were not receiving disease-modifying therapies (DMT) at the time of blood collection. To generate LCL, PBMCs were resuspended in Panserin 401 medium (PAN-Biotech GmbH, Aidenbach, Germany) supplemented with penicillin-streptomycin (Life Technologies, Carlsbad, CA, USA). PBMC were seeded at a density of 3 × 10^5^ cells per well in a 24-well plate. EBV-containing supernatant was collected from the B95.8 cell line and added at a ratio of 1:1 per well. Cells were incubated at 37 °C in a 5% CO_2_ humidified atmosphere. After 2 weeks, the conditioned medium was replaced with fresh medium. Once a week the cells were fed with fresh medium. After 5–6 weeks, big lymphoblastoid colonies had grown and could be expanded. Typically, the cells were split twice a week, at a ratio of 1:2, and harvested for downstream experiments 4–5 weeks after the first split. 

### 2.2. Flow Cytometry

Surface antigen expression was detected by flow cytometry. Cells were resuspended in ice-cold PBS with 2 mM EDTA and stained for 30 min at 4 °C using 5 µL of two-color antibody solutions αCD45-FITC/αCD14-PE, αCD3-FITC/αCD19-PE, αCD3-FITC/αHLA- DR-PE, or αIgG1-FITC/αIgG2a-PE from the BD Simultest IMK Plus reagent kit, single-color antibodies αCD23-PE or αIgG1-PE control (all from BD Biosciences, Franklin Lakes, NJ, USA). After washing with 1 mL PBS/EDTA solution, cells were analyzed on a LSRII cytometer using FACSDiva software version 8.0.1 (BD Biosciences, Franklin Lakes, NJ, USA).

### 2.3. RNA Extraction, cDNA Generation, and Quantitative Real-Time PCR 

Total cellular RNA was isolated using the NucleoSpin RNA kit (Machery-Nagel GmbH & Co. KG, Düren, Germany) following the manufacturer’s instructions. Transcription into cDNA was performed using 1 μg total RNA in 16 μL nuclease-free water and 4 μL qScript cDNA 5× SuperMix (QuantaBio, Beverly, MA, USA). For qRT-PCR, each reaction contained 0.5 µL of cDNA, 500 nM of forward and reverse primer, 5 µL of PowerUP SYBR Green 2× Master Mix (Applied Biosystems by Thermo Fisher Scientific, Waltham, MA, USA), and 4 µL of nuclease-free water. All primers are listed in Table 1.

The amplification protocol included an initial denaturation step at 95 °C for 10 min, followed by 40 cycles with denaturation at 95 °C for 15 s, primer annealing, amplification, and extension at 61 °C for 60 s. Two technical replicates were measured for each sample. LCL from the same PBMC donor were taken together and therefore served as biological replicates. A total of 30 PBMC and 94 LCL were analyzed from 32 individuals with MS, as well as 10 PBMC and 29 LCL from 10 controls. The analysis was performed using QuantStudio3 and QuantStudio Design and Analysis Software v.1.4.3 (Applied Biosystems by Thermo Fisher Scientific, Waltham, MA, USA). For copy number determination, standard curves were prepared using dilutions from 10 ng to 10^−11^ ng of purified target DNA. The copy number was calculated with respect to the molecular weight of the target DNA amplicon (EBNA-1: 156.381 kDa; EBNA-2: 247.219 kDa; HERV-K GAG: 103.226 kDa; HERV-W1 ENV: 84.057 kDa) using the DNA Molecular Weight calculator at https://www.bioinformatics.org/sms2/dna_mw.html (accessed on 1 January 2020). Standard curves can be found in the supplement (Supplementary Figure S1).

### 2.4. RNAseq Analysis

Generation of RNAseq data was performed by Novogene UK Co., Ltd. (Cambridge, UK) using the Illumina Novaseq6000 system, and can be downloaded from the NCBI Short Read Archive (SRA) under BioProject PRJNA765133. All samples presented in this manuscript passed the quality control. The quality control report is provided in the supplement. A total of 16 PBMC and 79 LCL from 32 individuals with MS (n_LCL_ = 59 and n_PBMC_ = 9) and 10 controls (n_LCL_ = 20 and n_PBMC_ = 7) were analyzed. LCL from the same PBMC donor were taken together and therefore served as biological replicates. Additional RNAseq data from activated B cells were downloaded from SRA (BioProjects PRJNA397793 [42] and PRJNA702159 [43]). Quantification of overall HERV expression was performed as described [44] by using the Galaxy server [45] for Bowtie2 mapping and quantification by FeatureCounts [46]. Quantification of human transcriptome data was performed by Novogene UK Co., Ltd. (Cambridge, UK) using human genome version GRCh38/hg38. For all analyses, fragments per kilobase per million (FPKM) values were calculated to quantify and normalize the counted fragments with FeatureCounts, according to the transcript length and the total number of reads.

To identify differentially expressed genes, we determined the fold changes calculated from the median of one group divided by the 75th percentile of the other group. Heatmaps of strongest differentially expressed genes were performed using the heatmap webtool at http://heatmapper.ca/expression/ (accessed on 1 August 2022). For all volcano plots, GraphPad Prism was used (version 8.3.0 for Windows, GraphPad Software, San Diego, CA, USA).

To study the influence of environmental factors and clinical parameters (as, e.g., the relapse rate), the paired-end reads of the sequence data were mapped to a small synthetic virus metagenome using Bowtie2 (Galaxy Version 2.4.2 + galaxy0) from the Galaxy platform [45] with default settings. We have added the fasta file of this genome, including selected sequences of housekeeping genes, endogenous and exogenous viruses, in the supplement (Viruses21B4.fasta). For quantification of gene expression from the BAM files, the mapped reads were counted using FeatureCounts (Galaxy Version 2.0.1 + galaxy2 [46]). The GTF file used for quantification (Viruses21B4.gtf) is provided in the supplement. We used the following settings: enable counting of fragments instead of reads (-p), only allowing fragments with both reads aligned (-B), enable both multi- and overlapping features (-M -O). This has the advantage, that the readthrough transcripts in the smaller exons (F/Q and U) can also be detected and would not only be counted, e.g., for the larger EBNA-1 K exon. This provides the highest probability of detecting all lytic EBNA-1 transcripts starting in the F/Q promoter region.

To identify MS biomarkers within our LCL model, differentially up-regulated genes in MS (*p* < 0.05) were identified, and for each gene the 16th highest FPKM value in MSLCL (n = 59) was determined. Subsequently, the genes that showed an expression below this threshold in all coLCL (n = 20) were identified. As a result, we obtained a list of genes that had an expression above a target-dependent threshold in at least 25% of MSLCL. This cut-off was never exceeded by any coLCL. To identify targets that correlated with the relapse rate in MS, we used MSLCL in at least duplicates from each seven individuals with (n = 14) and without relapses (n = 15) in the last two years matched by gender and the duration of disease ±1 year. Due to the limited number of samples, we determined targets that had an expression above or below a cut-off value in at least 75% of the MSLCL from individuals with relapses using the 11th highest FPKM.

### 2.5. Statistics

For all statistical analyses, GraphPad Prism was used (version 8.3.0 for Windows, GraphPad Software, San Diego, CA, USA). Comparisons of two groups were performed using *t*-test. To compare more than two groups, a one-way ANOVA with Dunnett T3 post-hoc test was performed. Multiple comparison analyses were performed using two-way ANOVA, followed by Bonferroni multiple comparisons test. The *p* values are indicated by asterisks: **** *p* < 0.0001, *** *p* < 0.001, ** *p* < 0.01, * *p* < 0.05. To determine the specificity and the sensitivity of putative biomarkers, receiver operating characteristic (ROC) analyses were performed.

## 3. Results

### 3.1. The GAG Regions of HERV-K (HML-2) Loci Are Up-Regulated by EBV-Triggered Immortalization

We studied the expression of EBV and distinct HERVs, which are frequently suggested to play a role in the pathogenesis of MS. Therefore, we designed a small virus meta-genome including the sequence of EBNA-2 as well as EBNA-1 transcripts including the exons F/Q, U and K, as well as sequences from HERV-W and HERV-K family members. Regarding HERV-W family, we focused on the envelope from ERVWE-1 coding locus on chromosome 7 (HERV-W1 *ENV*) and another envelope with a yet unknown genomic location named MS-associated retrovirus (MSRV *ENV*). Further, we investigated the expression of a group-specific antigen (GAG) region of HERV-K4 belonging to the biologically most active HERV-K (HML-2) family. Besides RNAseq, we determined copy numbers of the same HERV-W1 *ENV*, EBNA-2 and lytic EBNA-1 transcripts by qRT-PCR, using a larger number of PBMC samples and multiple LCL per donor. In total, 40 PBMC and 123 LCL from 32 individuals with MS (n_LCL_ = 94 and n_PBMC_ = 30) and 10 controls (n_LCL_ = 29 and n_PBMC_ = 10) were analyzed. Of notice, expression of HERV-K *GAG* was studied using primers that detected not only *GAG* from HERV-K4, but also other members of the HERV-K (HML-2) family.

**Figure 1:**
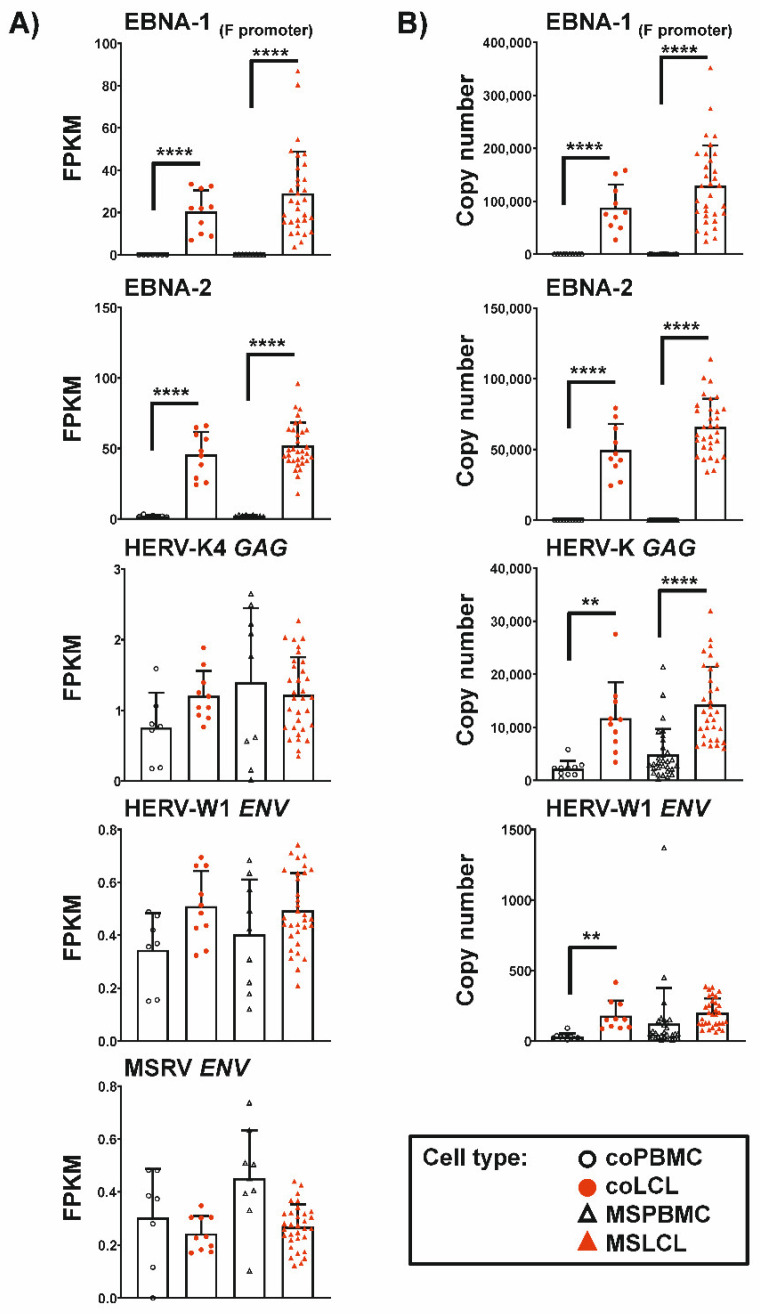
Comparison of EBV and HERV expression in PBMC and after EBV-triggered immortalization of B cells. The graphs show mean ± SD of FPKM of RNAseq analyses (**A**) or copy numbers of qRT-PCR analyses (**B**). LCL from the same individual served as biological replicates. Statistics: One-way ANOVA with Dunnett T3 post-hoc test; **** *p* < 0.0001, ** *p* < 0.01.

Comparing the expression in PBMC and LCL, we observed strong increase after EBV-triggered immortalization of both EBNA-2 (*p* < 0.0001) and lytic EBNA-1 (*p* < 0.0001) using RNAseq and qRT-PCR (Figure 1). No expression of the EBV transcripts was observed in any of the PBMC samples studied. In contrast, the expression of the HERVs analyzed could be detected in LCL and PBMC, though at low levels. In controls, HERV-W1 *ENV* showed higher expression in LCL than in PBMC with significant difference in qRT-PCR analyses. In individuals with MS, high HERV-W1 *ENV* levels were also observed in several PBMC samples without significant up-regulation in LCL. However, the overall mean expression was comparably elevated in LCL from both controls and individuals with MS. For MSRV *ENV*, the expression showed no significant changes after EBV-immortalization by RNAseq analyses. Further, the expression of HERV-K4 *GAG* showed no significant differences in PBMC and LCL from individuals with MS or controls. In qRT-PCR analyses targeting broad numbers of HERV-K (HML-2) family members due to their high sequence homology, HERV-K *GAG* transcription was up-regulated after EBV-induced immortalization of both MS (*p* < 0.0001) and control (*p* < 0.01) samples.

### 3.2. Transcription of EBNA-1 from the Lytic Promoter Is Negatively Correlated with Age of PBMC Donors

Next, we investigated the influence of environmental risk factors and clinical para-meters on the expression of the mentioned EBV and HERV targets in LCL from individuals with MS. We observed increased levels of lytic EBNA-1 transcripts (*p* < 0.01) in LCL from individuals with MS treated with natalizumab compared to those not receiving disease-modifying therapies (Table 2). The primers used for EBNA detect transcripts initiated at the F promoter that is used during EBV lytic cycle. Consequently, detection of EBNA-1 transcripts with these primers in LCL correlates well with the B cell-immortalization capacity of culture supernatants from these cells [38].

Of interest, EBNA-1 transcripts were increased (*p* < 0.01) in individuals without relapses compared to individuals with one or two relapses in the last two years. Further, EBNA-1 expression was 1.7-fold higher in individuals with low disability scores (EDSS < 2), although this increase was not significant. Interestingly, the expression of EBNA-1 was negatively correlated with age (r = −0.36). Using qRT-PCR analyses, we could not observe significant differences in the relapse rate, the treatment with natalizumab or the EDSS score, but EBNA-1 expression also tended to be higher in individuals without or with minimal disabilities compared to those with higher EDSS scores (Supplementary Table S1). The difference in EBNA-1 between the younger to the elder MS subgroups could be confirmed (*p* < 0.001 and *p* < 0.0001) using qRT-PCR analyses in 94 LCL from 32 individuals with MS. In addition, we detected higher expression of both EBNA2 (*p* < 0.05) and EBNA1 transcripts (*p* < 0.05) in MSLCL compared to controls. Further, the up-regulation of all EBV and HERV transcripts by qRT-PCR was more pronounced in MSLCL established from female donors compared to gender-matched controls, while no difference in expression could be detected in the male counterparts (Supplementary Figure S2). However, these differences in EBV and HERV expression with gender were not significant in RNAseq analyses (Supplementary Figure S3). For HERV-K *GAG*, differences in gene expression might be explained by targeting more than one distinct transcript due to the high similarity within the HERV-K family. For all additional investigated environmental sequence features and clinical parameters, no significant changes in expression were observed.

### 3.3. Differential Expression Pattern in Human Transcriptome in LCL from MS-Derived Samples and Controls

**Figure 2:**
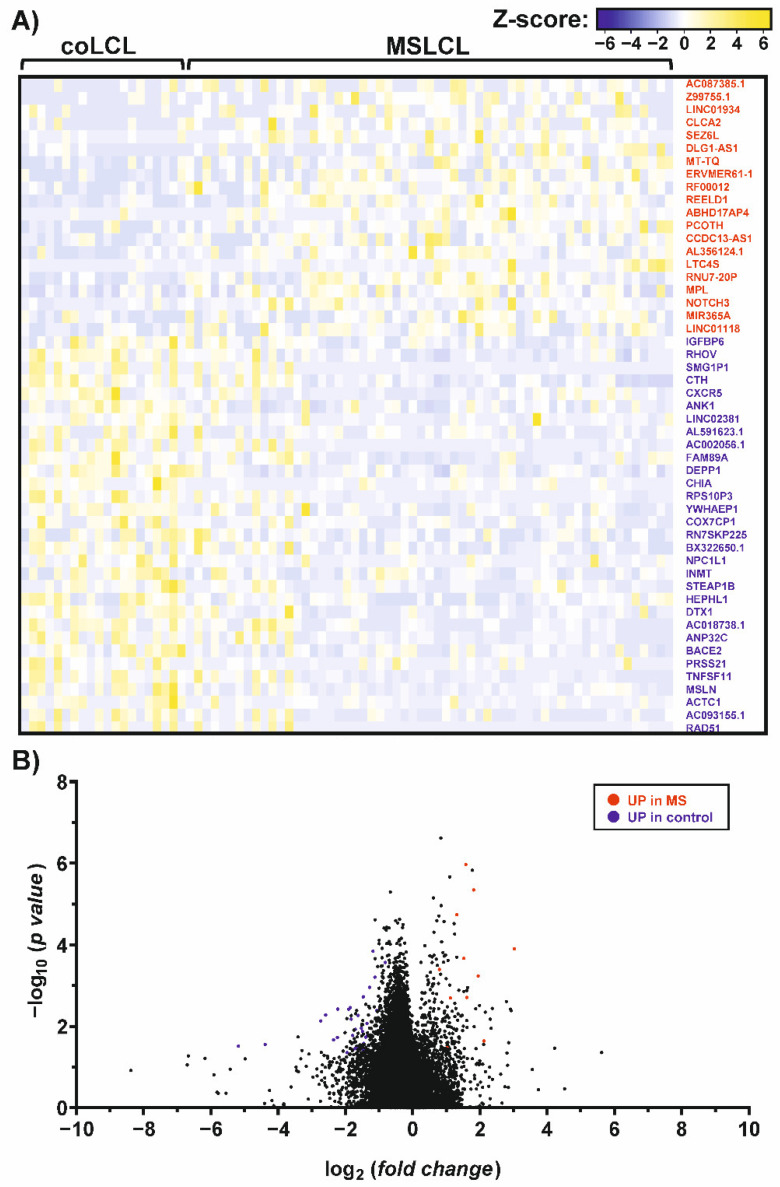
Differential gene expression patterns in individuals with MS and controls, assessed by RNAseq analysis. The graphs represent a heatmap (**A**) and a volcano plot (**B**) of differentially expressed genes (fold change > 1.5, *p* < 0.05) in MS and controls. Up-regulated genes in MS (red) and controls (blue) were indicated and ranked from highest to lowest fold change in MSLCL. For this analysis, a total of 20 LCL established from controls (coLCL) and 59 LCL from individuals with MS (MSLCL) were used. Human genome version GRCh38/hg38 was used for RNAseq read mapping. Fold changes and statistical significances were listed in the supplement (Supplementary Tables S3 and S4).

Mapping against the human genome version GRCh38/hg38 showed that gene expression profiles of PBMCs and LCL are distinctly different, as expected. Well-known EBV-induced genes like EBI3 and CD70 were up-regulated 150- to 160-fold (*p* < 0.0001), in both coLCL and MSLCL. Furthermore, cytokines related to cell growth checkpoints, e.g., CCL22 and CCL25, were observed in the LCL (Supplementary Table S2). In PBMCs, genes that are mainly expressed by T lymphocytes, like CD3D (fold change: 503.1) and CD7 (fold change: 599.9), or monocytes, such as CD14 (fold change: 1610.9), were among the 100 most up-regulated genes compared to the LCL as pure B cell lines verified by flow cytometry (Supplementary Figure S4). In contrast, the overall gene expression pattern was highly similar in the comparison of MSLCL and coLCL, resulting in a limited number of differentially expressed genes (Figure 2). Notably, an ERV element of the medium reiteration frequency (MER) family, ERVMER61-1, was included within the 20 up-regulated genes (fold change > 1.5, *p* < 0.05) in MSLCL. Furthermore, *REEDL1*, *RNU7-20P*, *RF00012* (member of U3 snoRNA family), *MT-TQ*, *DLG-AS1* and *MPL* were increased most significantly in MSLCL (STab. 3). In coLCL, 39 genes were found to be up-regulated (fold change > 1.5, *p* < 0.05). The decidual protein induced by progesterone 1 autophagy regulator (*DEPP1*) was increased most significantly in coLCL compared to MSLCL, which might suggest higher activation of autophagy mediated by oxidative stress [47]. Furthermore, beta-secretase 2 (*BACE2*) which cleaves the amyloid precursor protein (APP) in the Aβ domain, preventing Aβ generation, e.g., in Alzheimer’s disease [48], as well as *RHOV, CTH, ANK1, HEPHL1, DTX1, DHX9P1, TNFSF11* and *AC018738.1* showed strongest evidence of differential expressions in controls (Supplementary Table S4).

### 3.4. Up-Regulation of Distinct HERV-K Loci and ERV3-1 in LCL from MS-Derived Samples

**Figure 3:**
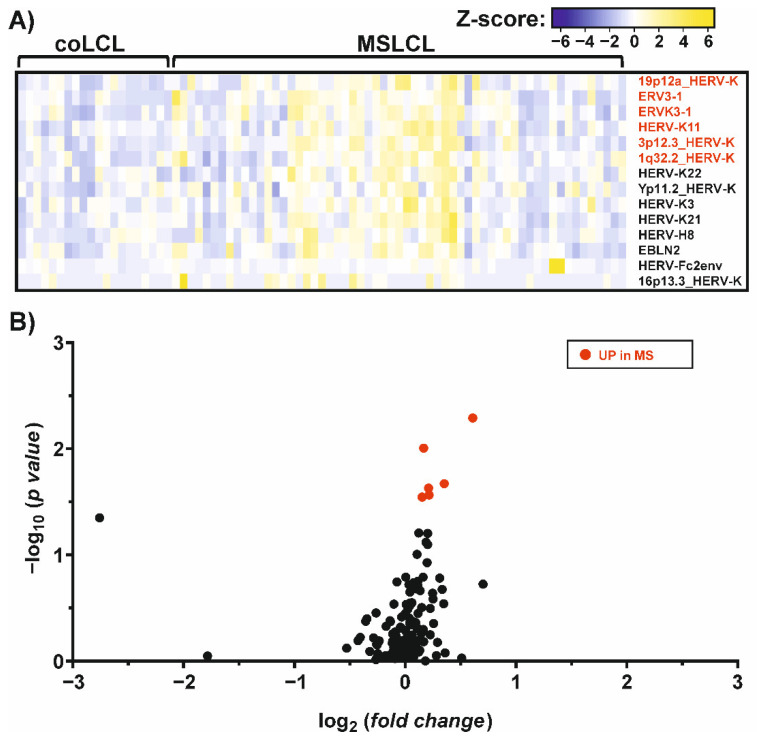
Differential expression of endogenous virus elements in MS and controls, assessed by RNAseq analysis. The graphs represent a heatmap (**A**) and a volcano plot (**B**) of differentially expressed genes (*p* < 0.05) in MS and controls. Up-regulated genes (fold change > 1) in MS (red) were indicated and ranked from highest to lowest fold change in MSLCL. None of the ERVs analyzed were found to be significantly up-regulated in control samples. For this analysis, a total of 20 LCL established from controls (coLCL) and 59 LCL from individuals with MS (MSLCL) were used. A synthetic virus metagenome was used for RNAseq read mapping. Fold changes and statistical significances were listed in the supplement (Supplementary Table S6).

To investigate the increase in HERVs in LCL and especially in MS samples, we mapped the reads against a synthetic virus metagenome including more than 100 individual HERV sequences [44]. As also exogenous viruses, like EBV, were included in this mapping process, we observed a distinct increase in EBNA-1 in LCL compared to PBMCs as expected. Among the differentially expressed HERV elements, HERV-K18, was most increased with a fold change of 2.1. Additionally, six HERV elements were found to be up-regulated after the EBV-triggered immortalization of B cells (Supplementary Table S5): three HERV-K elements located at chromosome 1q21.3 (JN675012.1), 12q24.11 (JN675069.1) and 22q11.23 (JN675088.1), as well as HERV-KC4 (X80240.1), HERV-9 (X57147.1) and HERV-W5 (AC117456.6:51035-52536). Interestingly, the up-regulation of these HERVs can be observed in B cell receptor (BCR) and CD40-stimulated B cells [42], but not in B cells stimulated by phorbol myristate acetate (PMA) [43] (Supplementary Figure S5), suggesting activation of HERVs in B cells by stimulation of the BCR/CD40, which are pathways used by EBV for immortalization. Comparing MS and controls, we observed only six HERVs that showed an increased expression in MSLCL (Figure 3). Of interest, five of these HERVs belong to the HERV-K family: ERVK3-1 (NC_000019: 58305374-58315657), HERV-K11 and three elements located at chromosome 19p12a (JN675076.1), 3p12.3 (JN675019.1) and 1q32.2 (JN675016.1). Furthermore, ERV3-1 (NC_000007.14: 64990356-65006687) belonging to the HERV-R family was up-regulated most significantly in MSLCL (Supplementary Table S6). In contrast, no HERV sequence was significantly up-regulated in LCL from controls.

### 3.5. Discovery of Gene Panels for MS and Relapse Risk Using RNAseq Data from LCL

To identify target genes that might be used as possible MS biomarkers, we analyzed the previously mentioned RNAseq data mapped against the human genome GRCh38/hg38, and the synthetic virus transcriptome for genes that were highly expressed at least in subgroups of MSLCL but not in coLCL. Among all up-regulated genes in MSLCL, we observed 35 genes showing a high expression level in at least 25% of the MSLCL which was never exceeded by any coLCL (Table 3).

**Figure 4:**
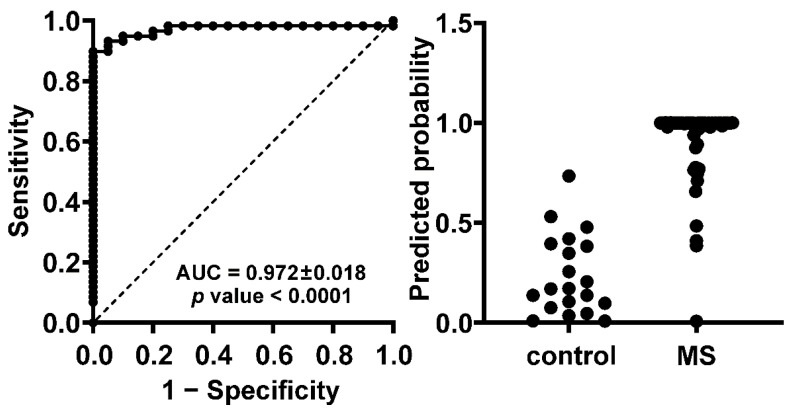
A panel of 15 target genes has high predictive power distinguishing between MS and control samples. RNAseq data of 20 LCL from 10 controls and 59 LCL from 32 individuals with MS were analyzed. The presented ROC curve and prediction probability were performed within a multiple logistic regression analysis using GraphPad PRISM 8.3.0. The 15 genes included for this analysis are listed in Table 3 with adjusted *p* values below 0.05. For ROC curve analysis, area under the curve (AUC) ± SE is indicated. A positive predictive power of 100% could be achieved using a classification cut-off at 0.75, suggesting no false positive predictions and a correct identification of about 88% of MSLCL.

We observed that one MSLCL did not show increased expression above the cut-off for any of these genes. However, this MSLCL had a duplicate LCL from the same subject (MSLCL-030) which did show increased expression above the cut-off for 8 of the 35 genes. Variability was also observed within the LCL from other subjects, suggesting that the usage of multiple LCL from one donor yields more powerful gene expression analyses (Supplementary Table S7). Using LCL duplicates, all 32 individuals with MS were identified with increased expression even for the smaller 15 gene panel, which we have chosen using multiple comparison analysis from all 35 targets (adjusted *p* < 0.05). The ROC curve from this smaller MS gene panel confirmed the high predictive power with an AUC of 0.972 ± 0.018 (Figure 4).

Second, we analyzed for targets that were correlated with the relapse rate in MS. Therefore, we used LCL from each 7 MS individuals with and without relapses matched by gender and duration of the disease ±1 year. We identified two genes above and six genes below (*p* < 0.05) a target-specific threshold in at least 75% of the LCL from individuals with relapses (Table 4). All individual ROC curves from the MS risk targets and the genes correlating with relapse rate can be found in the supplement (Supplementary Figures S6 and S7).

## 4. Discussion

In the present study, we investigated differential gene expression patterns in MS and controls after EBV infection of B cells, as EBV infection is a major risk factor for the subsequent development of MS [5,6,7,8,9,10,11,12,13,14,15]. To investigate the influence of environmental risk factors and clinical parameters, we studied selected EBV and HERV targets in LCL by RNAseq and qRT-PCR. For lytic EBNA-1, we observed differential expression with certain parameters investigated. Using RNAseq, EBNA-1 showed up-regulation in MSLCL from individuals treated with natalizumab compared to those not receiving disease-modifying therapies, and in individuals without relapses compared to individuals with one to two relapses in the last two years. However, both results regarding the relapse rate or the DMT could not be confirmed using qRT-PCR method (Supplementary Table S1, Supplementary Figure S9 and S10). Additionally, our results from qRT-PCR suggested an up-regulation of EBNA-1, EBNA-2 and HERV-K *GAG* expression, which was more pronounced in LCL from female individuals with MS. Differential expression of HERV-K4 *GAG* was not detected by RNAseq analysis. Quantification of reads that mapped exclusively to the HERV-K4 region that was also amplified by PCR showed higher similarity with the qRT-PCR results (Supplementary Figure S8). For lytic EBNA transcripts we observed approximate reduction in expression levels by half from the youngest to the oldest group in both RNAseq and qRT-PCR analyses. While beneficial effects of vitamin D supplementation on inflammation in MS have been frequently observed [49,50], the contribution of vitamin D deficiency to MS risk, or its association with EBV in MS, is still elusive, and commonly conflicting results can be found in the literature [51,52]. According to the data presented, no significant differences in the studied EBV and HERV genes between deficient and non-deficient individuals could be observed in both, qRT-PCR and RNAseq analyses. However, the study presented here was limited to a single point in time of PBMC extraction. To confirm a relationship of EBV transcription and vitamin D deficiency in MS, we propose further investigation on EBV expression and the vitamin D titer at the stage of deficiency, and within time after successful vitamin D supplementation in LCL from individuals with MS and controls, as an interplay between EBV, HERVs and vitamin D seems at least plausible [53,54]. In this scenario, higher vitamin D titer after supplementation might result in reduced EBNA levels in EBV-immortalized B cells. For EBNA-2, the inverse correlation could be explained by overlapping DNA binding sites with the vitamin D receptor that would outcompete EBNA-2 at high vitamin D levels and vice versa [55]. Consequently, the transactivation of HERVs as putative EBV-triggered drivers in the pathogenesis of MS should be affected by lower EBV levels.

Regarding HERVs that can be transactivated by EBV, we observed an increase in HERV-K18 expression by up to 2.1-fold upon EBV-triggered immortalization. This result was not unexpected, as the Huber’s group has previously studied the mechanism of an EBV-triggered transactivation of HERV-K18. On resting B cells, EBV glycoprotein gp350 binds to CD21, and therefore leads to the activation of HERV-K18 ENV protein coding for a superantigen that strongly stimulates a large number of T cells [24,56,57]. In addition, we found that activation of HERV-K18 is not limited to the coding region of the ENV, as we observed an equal distribution of reads across the complete proviral sequence (data not shown).

On the other hand, the results for the expression pattern from HERV-W1 *ENV* and MSRV were more ambiguous. From the literature, one might expect that the expression of both MSRV *ENV* and HERV-W1 *ENV* RNA is up-regulated in B cells and monocytes after exposure to the EBV glycoprotein, gp350, that had been found to transactivate the ENV of HERV-K18 [58,59]. In our study, we used complete EBV from the B95.8 cell lines instead of gp350. However, we observed an increase in HERV-W1 *ENV* in controls after EBV-induced immortalization by qPCR, analyzing higher numbers of PBMC and multiple LCL per donor. This might be explained by high HERV-W1 *ENV* levels in PBMCs from several individuals with MS, which is supported by similar results from other studies suggesting an overall higher frequency of circulating HERV-W in individuals with MS [58,60]. Based on our data, we observed a tendency for higher HERV-W1 *ENV* expression which was not significant in PBMC of individuals with MS, but no up-regulation of MSRV *ENV* in LCL, although an increase in HERV-K18 and six other HERV loci occurred upon EBV-immortalization via CD40/BCR-receptor signaling pathway. Of interest, HERV-W5, belonging to the HERV-W family, was one of these EBV-activated HERV genes.

In the present study, we observed up-regulation of ERVMER61-1, several loci from HERV-K family and ERV3-1 exclusively in MSLCL. So far, the ERV3-1 element, belonging to the HERV-R family, was found to be differentially expressed in PBMCs from individuals with MS and controls [61] but none of the investigated ENV allelic forms were associated with MS [62]. The ERVMER61-1 was previously analyzed as a putative candidate lncRNAs in subtypes of breast cancer [63] and hepatocellular carcinoma (HCC), where it was thought to be included in a prognostic signature of dysregulated RNAs in HCC [64]. Of note, expression of these HERV loci did not show a dependency on age or gender (Supplementary Figures S11 and S12). Since the differences between MSLCL and coLCL were subtle, it should be discussed to what extent the expression level of HERVs alone is crucial in the pathogenesis of MS. HERVs are intrinsic transposable elements, which are capable of modulating their integration site within or near to genes. By disruption or promotion of co-localized genes, e.g., critical immune factors, the existence of full or partial HERV provirus sequences might have an impact itself [65,66]. As a result, HERV transcription is under constant surveillance by multilayered regulatory mechanisms of the host. This balance between the persistence of HERV expression and the host immune protection might be very subtle, especially in the case of HERV activation as a first line of antiviral defense counteracting infections with exogenous (retro-)viruses [67]. In the case of HERV glycoprotein expression, this mechanism of molecular mimicry could also accidently trigger other proteins with similarity in sequence that are principal targets of an autoimmune response, as it has been suggested for myelin and viral peptides from EBV and HERV ENV via binding to the MS risk factor HLA class II DR2b [68]. Recently, cross-reactive antibodies against EBNA-1 and the glial antigen GlialCAM have been found in the CSF of individuals with MS [69]. In addition, the EBV-specific T cell repertoire in individuals with MS seems to be expanded [70], and EBV-specific CD8-positive T-cells are frequently observed in the CSF and in brain tissue at sites of white matter lesions and the meninges in MS [71,72]. These observations suggest ongoing EBV (re-)activation that might challenge the immune homeostasis directly or by transactivation of HERVs. Besides the antiviral defense, HERVs seem to be involved in the maintenance of the pluripotency of stem cells [44] and are activated at specific developmental stages during embryogenesis and pregnancy, which also challenges the mentioned host–virus balance [73]. In summary, HERVs are able to regulate the host genes’ activity in several ways and at different expression levels. For future analyses, the HERV loci here identified should be considered as putative MS risk factors by, e.g., RNAseq analyses in a larger cohort. Considering the high variability in gene expression, we recommend including multiple LCL from each subject. Further, we suggest matching of MS and control samples by age and gender due to the tendencies we observed in the present study. A very recent report also found a slightly higher seroprevalence of EBNA-1 antibodies in females [74]. Finally, the expression of ERV3-1 should be also investigated on the protein level, as specific antibodies are available.

Besides HERV elements as candidate MS biomarkers, we identified a gene panel consisting of 15 targets that were highly expressed in at least 25% of our MS samples and could perfectly distinguish between our LCL from individuals with MS and the healthy controls, which never exceeded the given cut-off values for any target. Further, the same approach using multiple LCL from the same donor was used to explore targets that correlated with relapse risk. Despite a limited number of MS samples matched by gender and disease duration, we identified eight targets discriminating between our individuals with and without relapses. Of course, these data are preliminary and have to be examined with a larger number of samples, but this can be achieved through further collection and sharing of RNAseq data from LCL as we did in the SRA database from NCBI.

## 5. Conclusions

The collection of RNAseq data is increasing, and disease-associated biomarker panels might be a helpful diagnostic tool for more-extensive and individualized disease monitoring, as deep genomic analyses are becoming ever faster and less expensive. In this manuscript, we have presented a strategy for using common RNAseq analysis techniques to identify target genes that might be suitable as biomarkers for MS, or risk factors such as the relapse rate in an EBV-mediated B cell approach. Furthermore, our data suggest an EBV-triggered transactivation of HERVs with differential expression of ERVMER61-1, ERV3-1, and copies from the HERV-K (HML-2) family in LCL from individuals with MS.

## Figures and Tables

**Table 1:** Primers used for quantitative real-time PCR.

Target	Exemplary Accession Number	Sequence of Forward (f) and Reverse (r) Primer (5′-3′)
EBNA-1	V01555.2:62399-107964 [38]	f: GCTTTGCGAAAACGAAAGTG r: CCCCTCGTCAGACATGAT
EBNA-2	V01555.2:48888-49287 [39]	f: TCTGCTATGCGAATGCTTTG r: GAGGGTGCATTGATTGGTCT
HERV-K *GAG*	JN675025.1:1621-1787 [40]	f: GGCCATCAGAGTCTAAACCACG r: CTGACTTTCTGGGGGTGGCCG
HERV-W1 *ENV*	NM_014590.4:2206-2341 [41]	f: TGCTAACCGCTGAAAGAGGG r: CGAAGCTCCTCTGCTCTACG

**Table 2:** Influence of environmental and clinicopathological factors on EBV and HERV expression in LCL from MS individuals. RNAseq data from EBNA-1, EBNA-2, HERV-K4 *GAG*, HERV-W1 *ENV* and MSRV *ENV* were determined in a total of 59 LCL from 32 individuals with MS and presented as FPKM. LCL from the same individual served as biological replicates. Data are means ± SD. Statistics: Two-way ANOVA with Bonferroni’s posthoc test; *p* values < 0.05 from comparisons between the indicated groups (a: 1-2 relapses, b: no relapses; c natalizumab, d: no therapy) were indicated.

	EBNA-2	EBNA-1 F Promoter	HERV-K4 *GAG*	HERV-W1 *ENV*	MSRV *ENV*
GENDER					
Female (n = 21)	52.68 ± 18.79	32.69 ± 17.39	1.260 ± 0.4868	0.5098 ± 0.1336	0.2671 ± 0.0825
Male (n = 11)	50.72 ± 10.94	21.94 ± 22.88	1.145 ± 0.6285	0.4633 ± 0.1589	0.2747 ± 0.0856
SMOKING BEHAVIOR					
Smoker (n = 11)	58.75 ± 35.12	28.17 ± 24.38	1.275 ± 0.6163	0.5157 ± 0.1433	0.2326 ± 0.0830
Non-Smoker (n = 21)	50.75 ± 17.97	29.43 ± 17.55	1.192 ± 0.4969	0.4823 ± 0.1435	0.2891 ± 0.0768
VITAMIN D DEFICIENCY					
Deficient (n = 14)	50.27 ± 21.30	31.28 ± 20.28	1.303 ± 0.4520	0.5074 ± 0.1258	0.2613 ± 0.0677
Non-Deficient (n = 7)	46.47 ± 8.694	25.61 ± 13.12	1.153 ± 0.5582	0.4910 ± 0.1539	0.2620 ± 0.1024
Unknown (n = 11)	57.73 ± 11.50	28.24 ± 23.62	1.159 ± 0.6399	0.4783 ± 0.1649	0.2853 ± 0.0913
AGE AT STUDY ENTRY					
Age 20–30 years (n = 6)	48.70 ± 12.04	31.38 ± 28.13	1.044 ± 0.6815	0.4035 ± 0.1595	0.2604 ± 0.0483
Age 31–40 years (n = 12)	56.25 ± 18.99	37.85 ± 19.26	1.402 ± 0.4946	0.5590 ± 0.1213	0.3071 ± 0.0801
Age 41–50 years (n = 8)	46.37 ± 16.39	22.67 ± 13.87	1.165 ± 0.5178	0.4544 ± 0.1427	0.2646 ± 0.0794
Age 50–60 years (n = 6)	54.33 ± 15.15	17.34 ± 11.16	1.108 ± 0.4931	0.5064 ± 0.1266	0.2110 ± 0.0951
DURATION OF DISEASE					
Time > 10 years (n = 11)	51.44 ± 18.31	33.71 ± 21.30	1.218 ± 0.4479	0.5140 ± 0.1233	0.2618 ± 0.0986
Time ≤ 10 years (n = 21)	52.30 ± 15.70	26.52 ± 18.99	1.222 ± 0.5823	0.4832 ± 0.1526	0.2739 ± 0.0747
LIFETIME WITH DISEASE ^†^					
Lifetime ≥ 20% (n = 15)	51.66 ± 16.37	35.64 ± 23.88	1.282 ± 0.5405	0.5238 ± 0.1365	0.2726 ± 0.0860
Lifetime < 20% (n = 17)	52.31 ± 16.84	23.13 ± 13.43	1.166 ± 0.5357	0.4674 ± 0.1455	0.2672 ± 0.0814
RELAPSES IN THE LAST 2 YEARS					
Relapses > 2 (n = 7)	46.51 ± 15.05	27.71 ± 13.08	1.250 ± 0.5834	0.4629 ± 0.1424	0.2389 ± 0.0421
Relapses 1–2 (n = 17)	49.06 ± 13.71	22.35 ± 14.34 ^a^	1.041 ± 0.4737	0.4471 ± 0.1205	0.2722 ± 0.0903
Relapse free (n = 8)	63.09 ± 19.20	44.24 ± 27.20 ^b^	1.577 ± 0.4753	0.6201 ± 0.1192	0.2914 ± 0.0912
*p* value		^a^ vs. ^b^: *p* < 0.01			
DISEASE-MODIFYING THERAPIES (DMT)					
Natalizumab (n = 14)	49.40 ± 19.73	37.24 ± 21.63 ^c^	1.578 ± 0.3895	0.5632 ± 0.0361	0.2742 ± 0.0816
No DMT (n = 10)	56.70 ± 13.53	15.79 ± 10.82 ^d^	0.8971 ± 0.5186	0.4036 ± 0.1593	0.2671 ± 0.0794
Other therapies ^‡^ (n = 8)	50.70 ± 13.38	31.06 ± 17.93	0.9989 ± 0.4087	0.4852 ± 0.1046	0.2652 ± 0.0971
*p* value		^c^ vs. ^d^: *p*< 0.01			
EXPANDED DISABILITY STATUS SCALE (EDSS)					
EDSS 1.0–1.5 (n = 6)	48.13 ± 26.23	44.34 ± 33.14	1.252 ± 0.6962	0.4987 ± 0.1757	0.3046 ± 0.0996
EDSS 2.0–2.5 (n = 11)	55.21 ± 11.88	28.68 ± 14.78	1.211 ± 0.5583	0.4981 ± 0.1516	0.2803 ± 0.0668
EDSS 3.0–3.5 (n = 6)	53.58 ± 18.80	21.98 ± 15.75	1.137 ± 0.4647	0.4841 ± 0.1660	0.2963 ± 0.0741
EDSS 4.0–5.5 (n = 8)	50.27 ± 14.00	24.88 ± 12.94	1.277 ± 0.5465	0.4984 ± 0.1188	0.2132 ± 0.0835

^†^ Calculated from duration of disease divided by age at study entry. ^‡^ Interferon beta-1a, carbamazepine, alemtuzumab, glatiramer acetate, dimethyl fumarate, fingolimod (n = 3).

**Table 3:** Genes with higher expression in MSLCL samples. All genes with an increased expression according to RNAseq analysis in at least 25% of MS samples, never exceeded by any control (co) were listed. The AUC ± SE were determined and the FPKM at 100% specificity was taken as the cut-off value for predicting MS. Statistics: *p* values from unpaired *t*-test were adjusted by multiple correction using Bonferroni–Dunn method. The false discovery rate (FDR) was calculated by Benjamini–Hochberg method. Genes were ranked according to their adjusted *p* value.

Name	Mean (co) n = 20 [FPKM]	Mean (MS) n = 59 [FPKM]	*p* Value	Adjusted *p* Value (Bonferroni–Dunn)	FDR *q* Value (Benjamini–Hochberg)	AUC ± SE	Cut-Off [FPKM] at 100% Specificity
MT-TY	24.09	55.71	0.00003	0.0011	0.0006	0.790 ± 0.056	67.83
MT-TC	146.6	417.2	0.00004	0.0013	0.0006	0.847 ± 0.046	355.7
AL669831.1	0.1678	0.2549	0.00005	0.0017	0.0006	0.800 ± 0.052	0.277
MT-TE	4.012	9.250	0.00007	0.0024	0.0006	0.790 ± 0.053	10.39
LINC01529	0.0394	0.1054	0.00010	0.0034	0.0006	0.797 ± 0.052	0.119
AL365273.1	1.442	2.960	0.00010	0.0034	0.0006	0.773 ± 0.058	3.426
SNORA79B	0.2844	0.7127	0.00019	0.0065	0.0009	0.800 ± 0.053	0.807
MIR34AHG	0.4675	0.9528	0.00033	0.0116	0.0014	0.743 ± 0.062	1.189
ENTPD1-AS1	0.3070	0.5408	0.00037	0.0129	0.0014	0.761 ± 0.059	0.622
MFSD14C	0.7940	1.0780	0.00039	0.0137	0.0014	0.748 ± 0.060	1.263
RNU7-20P	0.2588	0.7819	0.00071	0.0247	0.0022	0.780 ± 0.055	1.016
REELD1	0.0213	0.0683	0.00076	0.0265	0.0022	0.791 ± 0.053	0.081
MT-TG	6.905	14.600	0.00088	0.0307	0.0022	0.734 ± 0.062	19.16
GABARAP	0.2457	0.5464	0.00088	0.0308	0.0022	0.757 ± 0.059	0.616
SNORD20	0.6918	1.5220	0.00107	0.0375	0.0025	0.747 ± 0.060	2.164
AL669831.3	0.0185	0.0411	0.00178	0.0622	0.0039	0.729 ± 0.063	0.049
RF00012	0.0564	0.1735	0.00219	0.0766	0.0045	0.775 ± 0.055	0.217
MPL	0.0084	0.0188	0.00234	0.0817	0.0045	0.726 ± 0.060	0.024
FAM89B	0.5908	0.8061	0.00743	0.2599	0.0137	0.701 ± 0.060	0.946
AC006001.3	0.5246	0.6044	0.00847	0.2963	0.0141	0.721 ± 0.058	0.657
EBF2	0.0007	0.0024	0.00849	0.2970	0.0141	0.696 ± 0.059	0.004
CFAP73	0.0359	0.0552	0.00998	0.3491	0.0159	0.699 ± 0.062	0.063
LINC01118	0.0050	0.0120	0.01080	0.3781	0.0164	0.682 ± 0.060	0.016
HERV-K 3p12.3	0.1862	0.2447	0.01254	0.4390	0.0177	0.637 ± 0.061	0.334
DLG1-AS1	0.0023	0.0103	0.01262	0.4416	0.0177	0.664 ± 0.063	0.016
DIAPH2-AS1	0.0130	0.0321	0.01402	0.4908	0.0189	0.713 ± 0.059	0.033
RPL41P1	133.8	317.3	0.01642	0.5746	0.0212	0.675 ± 0.062	276.9
MIR3150BHG	0.0372	0.1018	0.01693	0.5924	0.0212	0.680 ± 0.059	0.102
TTLL7-IT1	0.0022	0.0109	0.02014	0.7048	0.0243	0.704 ± 0.059	0.009
RNU6-32P	0.0636	0.1835	0.02500	0.8751	0.0292	0.670 ± 0.062	0.273
GOLGA6L4	0.0004	0.0041	0.02800	0.9798	0.0316	0.736 ± 0.055	0.002
HIST2H2AC	0.8899	1.21	0.06537	>0.999999	0.0654	0.606 ± 0.065	1.379
C3P1	0.0014	0.0045	0.02933	>0.999999	0.0321	0.659 ± 0.062	0.005
MIR221	0.4966	0.9103	0.03409	>0.999999	0.0351	0.614 ± 0.064	1.158
HERV-K11	0.2805	0.3140	0.03055	>0.999999	0.0324	0.683 ± 0.064	0.303

**Table 4:** Genes that correlated with relapse rate in LCL from individuals with MS. Each 7 MS individuals with or without relapses in the last 2 years were compared. They were matched by gender and the duration of disease ±1 year. LCL from these individuals were at least analyzed in duplicates by RNAseq. All genes with an exclusively up- or downregulated expression in at least 75% of MS samples with relapses in the last 2 years were listed. The AUC ± SE were determined and the FPKM at 100% specificity was taken as the cut-off value for predicting relapses. Statistics: *p* values from unpaired *t*-test were adjusted by multiple correction using Bonferroni–Dunn method. The false discovery rate (FDR) was calculated by Benjamini–Hochberg method. Genes were ranked according to their adjusted *p* value.

Name	Mean MS with Relapses in the Last 2 Years n = 14 [FPKM]	Mean MS w/o Relapses in the Last 2 Years n = 15 [FPKM]	*p* Value	Adjusted *p* Value (Bonferroni–Dunn)	FDR *q* Value (Benjamini–Hochberg)	AUC ± SE	Cut-Off [FPKM] at 100% Specificity
UP							
AK7	0.550	0.228	0.0001	0.0007	0.0004	0.895 ± 0.062	0.188
BX664727.3	0.0123	0.0004	0.0012	0.0105	0.0017	0.957 ± 0.043	0.006
DOWN							
ZNF302	1.904	2.942	0.0000	0.0003	0.0003	0.943 ± 0.041	2.237
PM20D2	1.224	2.598	0.0005	0.0046	0.0015	0.924 ± 0.053	1.410
ZNF283	0.293	0.471	0.0008	0.0068	0.0017	0.905 ± 0.059	0.347
DHFRP1	6.004	12.360	0.0009	0.0084	0.0017	0.871 ± 0.073	8.061
XKR9	0.037	0.085	0.0015	0.0135	0.0019	0.876 ± 0.071	0.044
ZNF195	1.658	2.670	0.0017	0.0153	0.0019	0.867 ± 0.075	1.787
TCERG1	4.837	6.280	0.0133	0.1195	0.0133	0.871 ± 0.078	4.994

## Data Availability

The datasets generated for this study can be found in the NCBI Sequence Read Archive (BioProject PRJNA765133) at https://www.ncbi.nlm.nih.gov/sra (accessed on 1 November 2022).

## Supplementary Materials

The following materials are available online at https://www.mdpi.com/article/10.3390/cells11223619/s1: Supplementary Figure S1: Standard curves relating the copy number of target genes to the cycle threshold (CT) measured by qRT-PCR. Supplementary Figure S2: Transcriptional activation of EBV and HERVs in MSLCL is pronounced in LCL generated from female donors (qRT-PCR). Supplementary Figure S3: Expression of EBV and HERVs in LCL from female and male PBMC donors (RNAseq). Supplementary Figure S4: Characterization of LCL from MS individuals (MSLCL) and control donors (coLCL) by flow cytometry. Supplementary Figure S5: The activation of HERV can be triggered through CD40 and B cell receptor (BCR)-signaling but not by phorbol myristate acetate (PMA) in B cells. Supplementary Figure S6: ROC curves of identified MS biomarkers. Supplementary Figure S7: ROC curves of targets that correlated with relapse rate in MS. Supplementary Figure S8: Comparison of HERV-K GAG expression in PBMC and EBV-immortalized B cells (RNAseq analysis). Supplementary Figure S9: Expression of EBV and HERVs in LCL from individuals with MS regarding disease-modifying therapy (DMT). Supplementary Figure S10: Expression of EBV and HERVs in LCL from individuals with MS regarding relapse rate. Supplementary Figure S11: Expression of HERVs up-regulated in MS regarding age of the individuals. Supplementary Figure S12: Expression of HERVs up-regulated in MS regarding gender of the individuals. Supplementary Table S1: Influence of environmental and clinicopathological factors on EBV and HERV expression in LCL from MS individuals. Supplementary Table S2: Strongest up-regulated genes in LCL. Supplementary Table S3: Strongest up-regulated genes in MSLCL. Supplementary Table S4: Strongest up-regulated genes in coLCL. Supplementary Table S5: Strongest up-regulated viral transcripts in LCL. Supplementary Table S6: Strongest up-regulated viral transcripts in MS-derived LCL. Supplementary Table S7: Variability of gene expression in LCL replicates from the same PBMC donor.
