# Supplementary material for: Epstein-Barr Virus-Induced Genes and Endogenous Retroviruses in Immortalized B Cells from Patients with Multiple Sclerosis
Source: Cells. 2022 Nov 15;11(22):3619. doi: 10.3390/cells11223619 (PMC9688211; doi:10.3390/cells11223619)
Supplement: Supplementary file 1 [file cells-11-03619-s001.zip › Supplementary_Data.pdf]

cells

MDPI

Supplementary materials

Epstein–Barr Virus-Induced Genes and Endogenous Retroviruses in Immortalized B Cells from Patients with Multiple Sclerosis

Lisa Wieland [1,2], Tommy Schwarz [1], Kristina Engel [2], Ines Volkmer [2], Anna Krüger [2], Alexander Tarabuko [1], Jutta Junghans [3], Malte E. Kornhuber [1], Frank Hoffmann [3], Martin S. Staege [2,*] and Alexander Emmer [1]

[1] Department of Neurology, Medical Faculty, Martin Luther University Halle-Wittenberg, 06120 Halle (Saale), Germany
[2] Department of Surgical and Conservative Pediatrics and Adolescent Medicine, Medical Faculty, Martin Luther University Halle-Wittenberg, 06120 Halle (Saale), Germany
[3] Department of Neurology, Martha-Maria Hospital Halle-Dölau, 06120 Halle (Saale), Germany
* Correspondence: martin.staege@medizin.uni-halle.de; Tel.: +49-34-5557-7280

This file contains:

Supplementary Figures

Supplementary Figure S1
Supplementary Figure S2
Supplementary Figure S3
Supplementary Figure S4
Supplementary Figure S5
Supplementary Figure S6
Supplementary Figure S7
Supplementary Figure S8
Supplementary Figure S9
Supplementary Figure S10
Supplementary Figure S11
Supplementary Figure S12

Supplementary Tables

Supplementary Table S1
Supplementary Table S2
Supplementary Table S3
Supplementary Table S4
Supplementary Table S5
Supplementary Table S6
Supplementary Table S7

Supplementary Figures

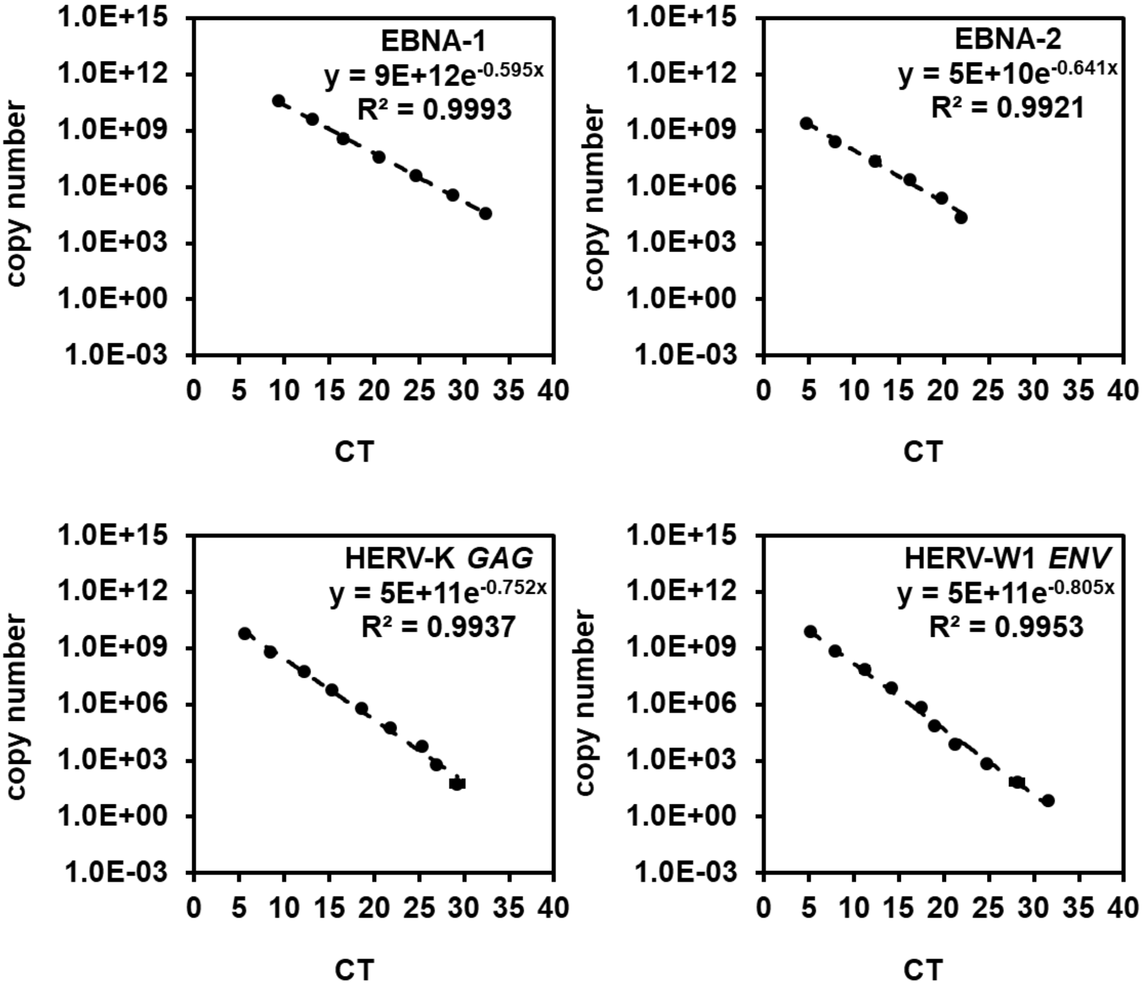

Supplementary Figure S1. Standard curves relating the copy number of target genes to the cycle threshold (CT) measured by qRT-PCR. For each target, dilution series from 10 ng to $1x10^{-11}$ ng of purified DNA were performed in triplicates. The graphs represent means±SD of data points belonging to the linear dynamic range (dashed line).

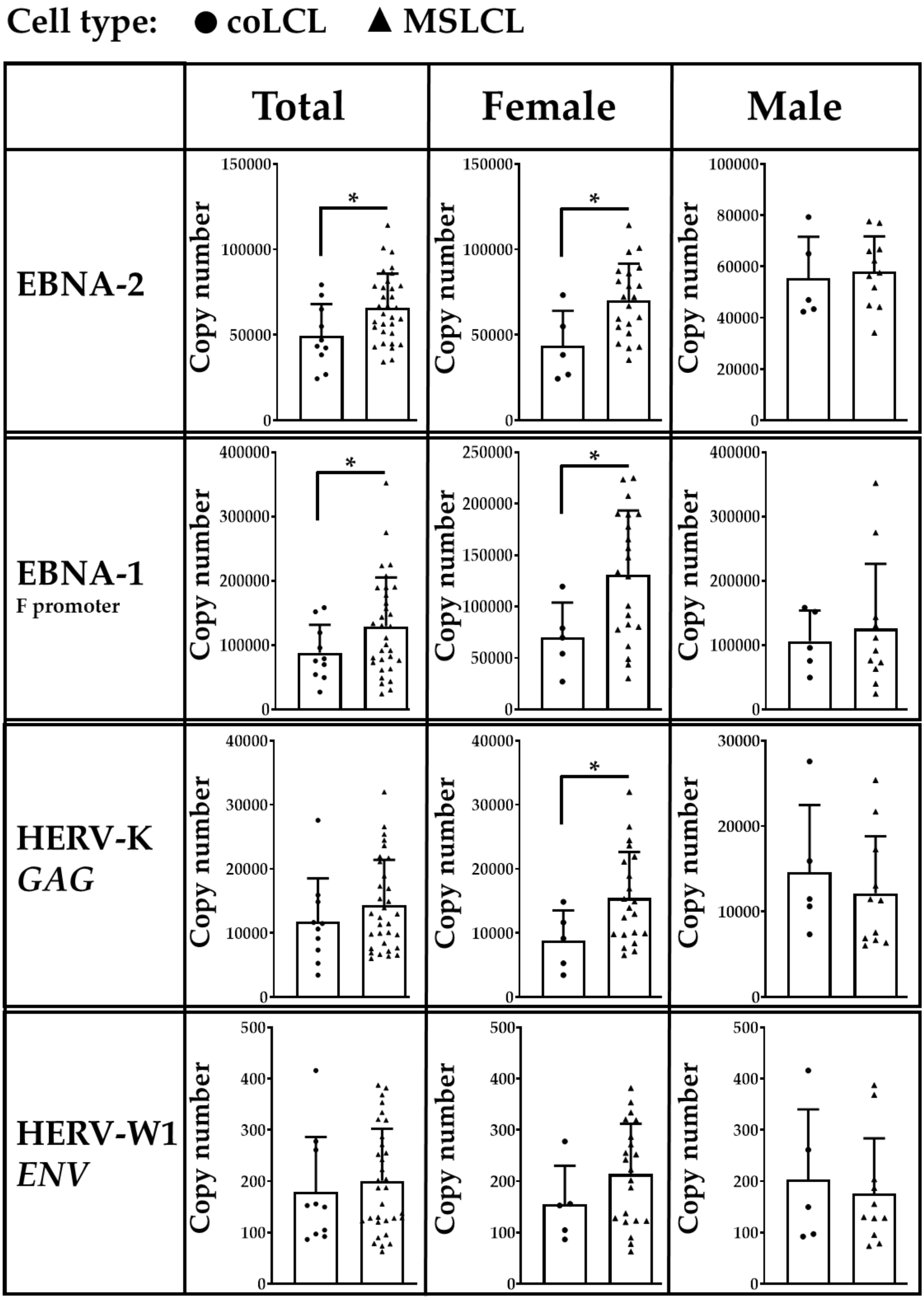

Supplementary Figure S2. Transcriptional activation of EBV and HERVs in MSLCL is pronounced in LCL generated from female donors (qRT-PCR). The graphs show means±SD of 94 LCL from 32 individuals with MS and 29 LCL from 10 controls analyzed by qRT-PCR. Copy numbers were determined by standard curves for individual target genes. Multiple LCL from the same donor served as biological replicates. The copy numbers from these LCL are also included in Figure 1B of the manuscript. Statistics: Welch's t-test; *$p < 0.05$.

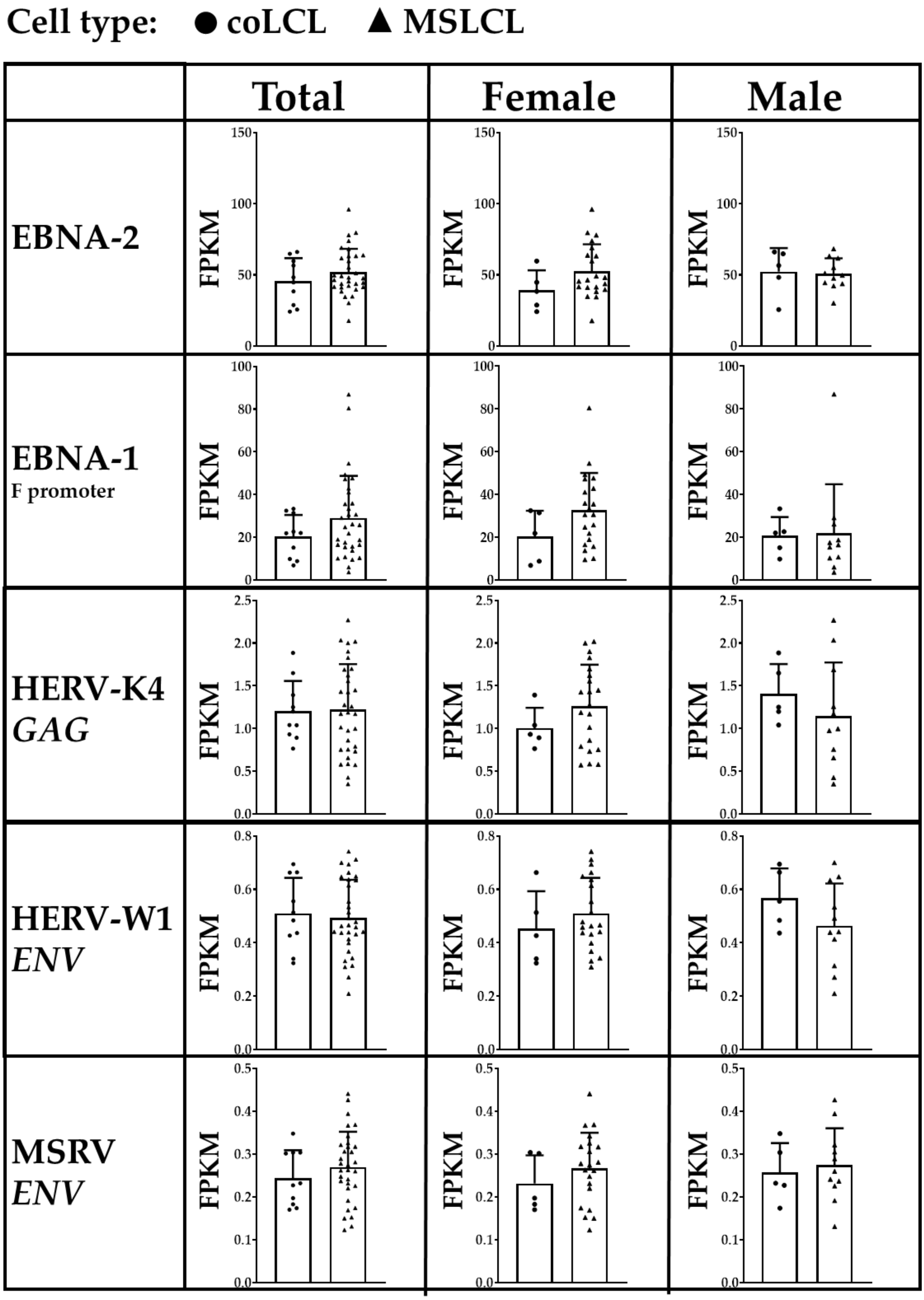

Supplementary Figure S3. Expression of EBV and HERVs in LCL from female and male PBMC donors (RNAseq). The graphs show means±SD of 59 LCL from 32 individuals with MS and 20 LCL from 10 controls analyzed by RNAseq. Multiple LCL from the same donor served as biological replicates. The RNAseq data from these LCL are also included in Figure 1A of the manuscript.

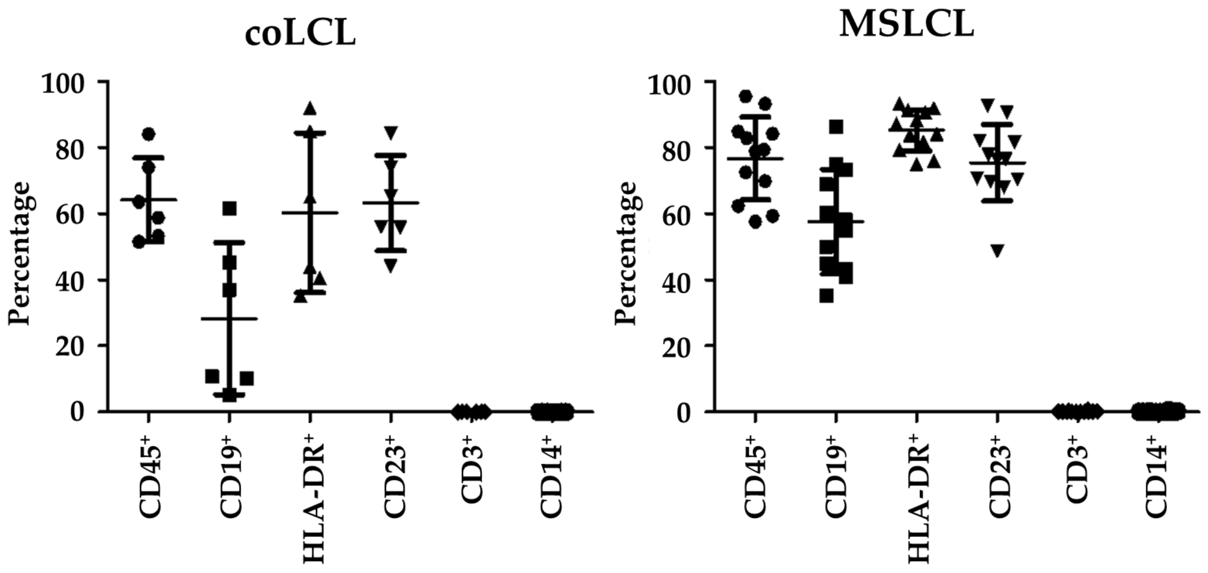

Supplementary Figure S4. Characterization of LCL from MS individuals (MSLCL) and control donors (coLCL) by flow cytometry. The graphs represent means±SD of the percentage of positive cells from all living cells. All tested coLCL and MSLCL showed expression of CD45, CD19, CD23, and HLA-DR. The presence of T-lymphocytes (CD3), as well as monocytes (CD14) was excluded.

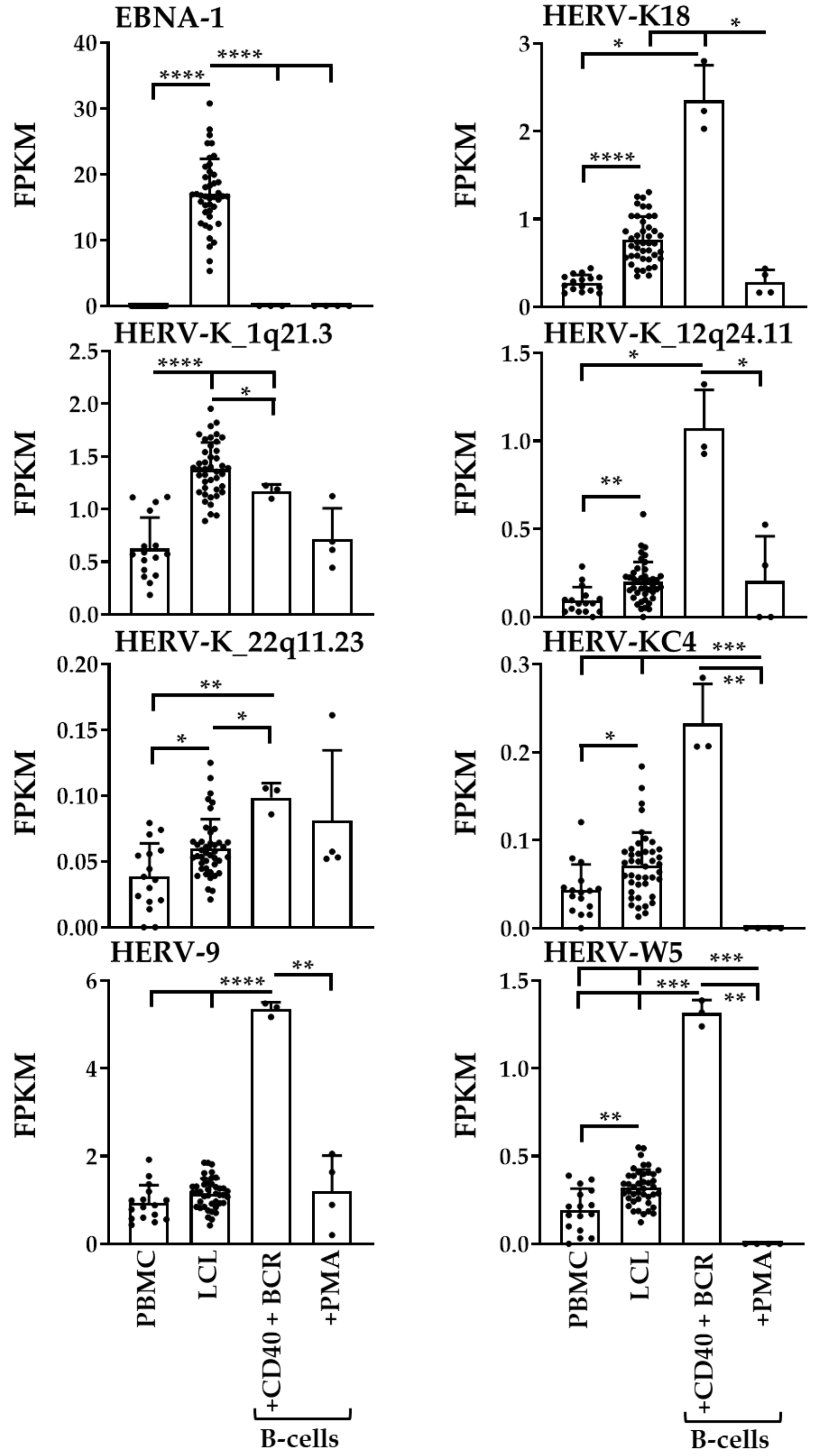

Supplementary Figure S5. The activation of HERV can be triggered through CD40 and B cell receptor (BCR)-signaling but not by phorbol myristate acetate (PMA) in B cells. The graphs show means±SD of FPKM from all PBMC (n = 16) and LCL (n = 42) in comparison to B cells stimulated with CD40 and BCR or PMA, respectively. The data from PBMC and LCL are the same as shown in Figure 1 of the manuscript. The presented HERV were selected according to their differential expression in PBMC and LCL as shown in the supplement (Supplementary Table S5). Statistics: one-way ANOVA with Dunnett T3 post-hoc test; ****$p < 0.0001$, ***$p < 0.001$, **$p < 0.01$, *$p < 0.05$.

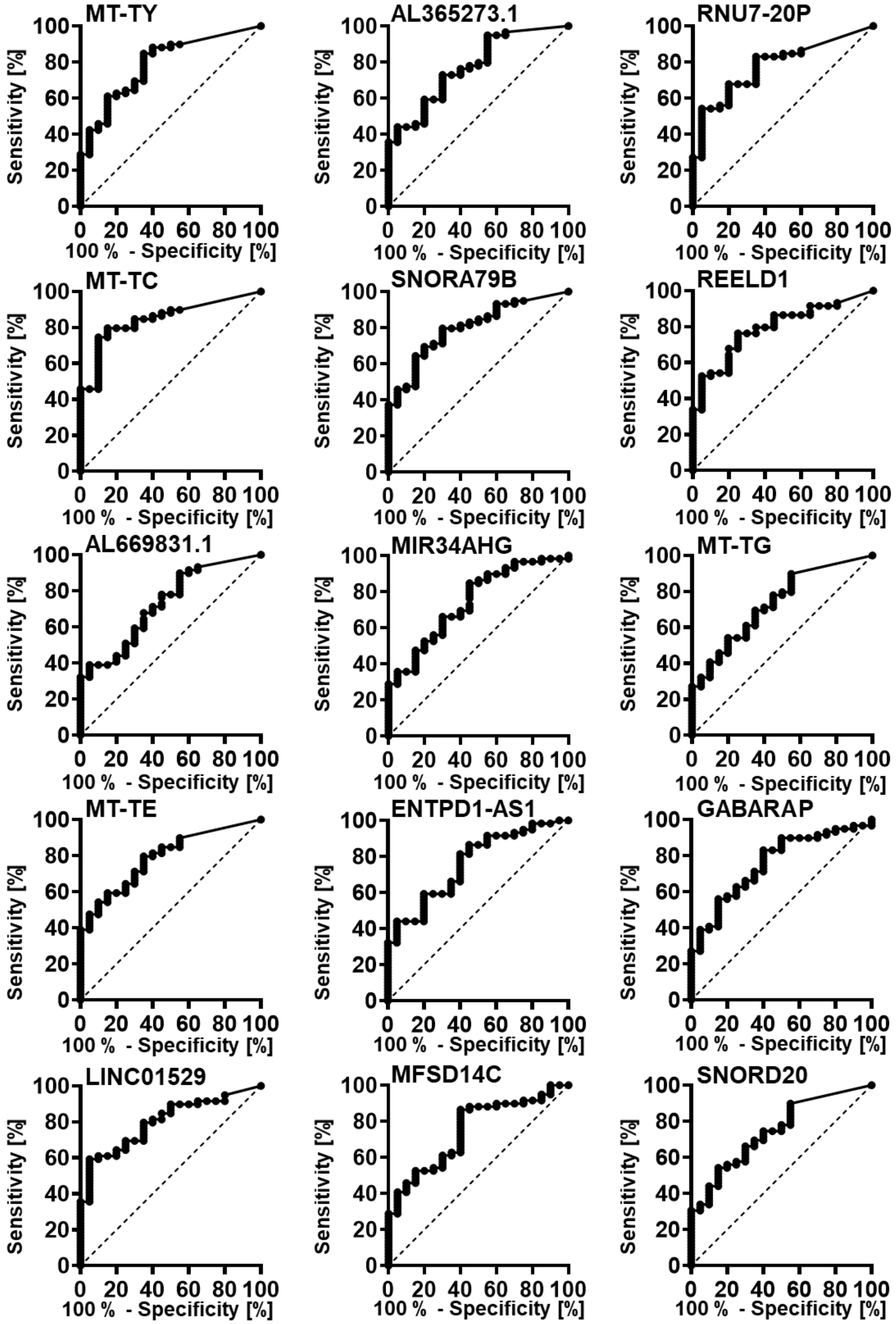

Supplementary Figure S6. ROC curves of identified MS biomarkers. Target genes were determined by RNAseq analyses of LCL as described in the manuscript. The ROC curve analyses were performed by GraphPad PRISM software version 8.3.0. The AUC of 0.5 is indicated by a dashed line.

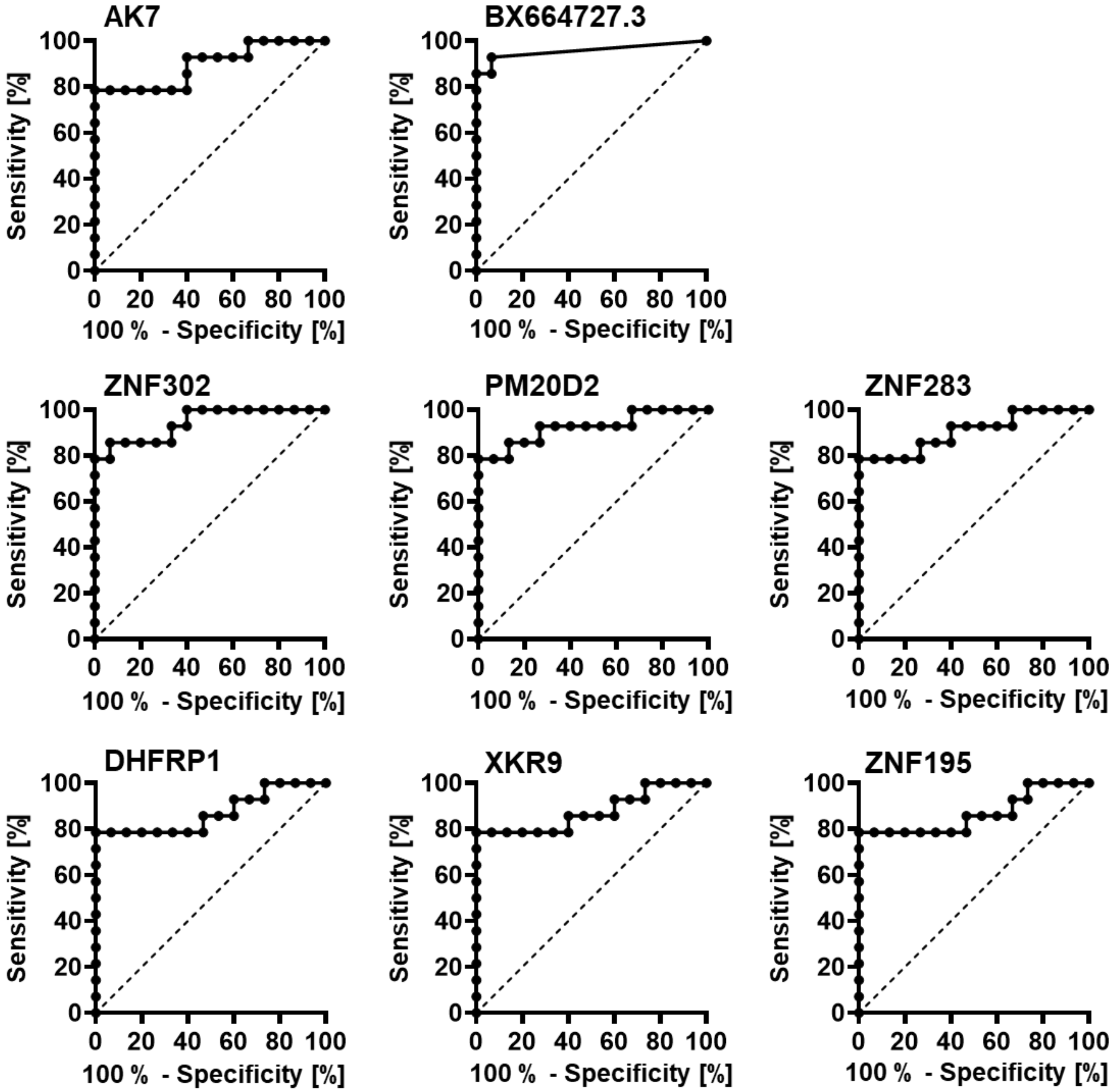

Supplementary Figure S7. ROC curves of targets that correlated with relapse rate in MS. Target genes were determined by RNAseq analyses of LCL as described in the manuscript. The ROC curve analyses were performed by GraphPad PRISM software version 8.3.0. The AUC of 0.5 is indicated by a dashed line.

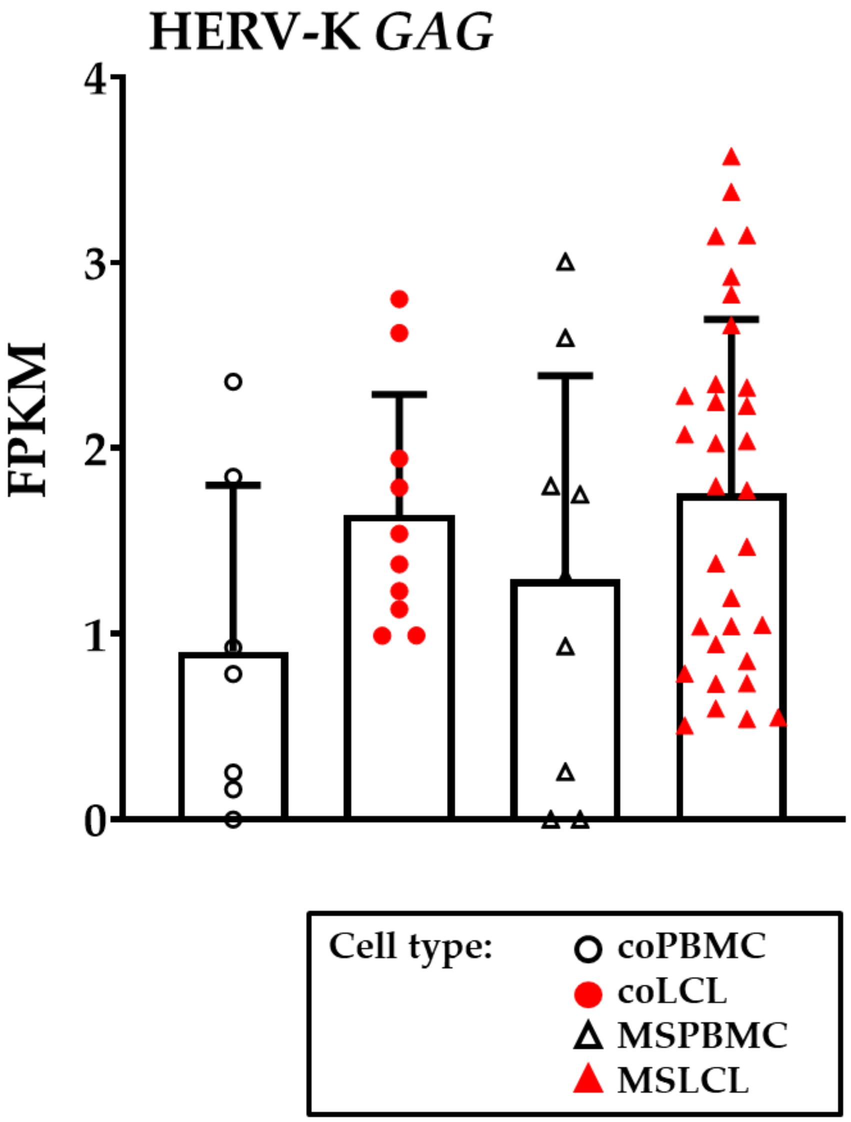

Supplementary Figure S8. Comparison of HERV-K *GAG* expression in PBMC and EBV-immortalized B cells (RNAseq analysis). Only reads that mapped to the region of the HERV-K4 *GAG* sequence that was the target for amplification by qRT-PCR were counted. Multiple LCL from the same donor served as biological replicates.

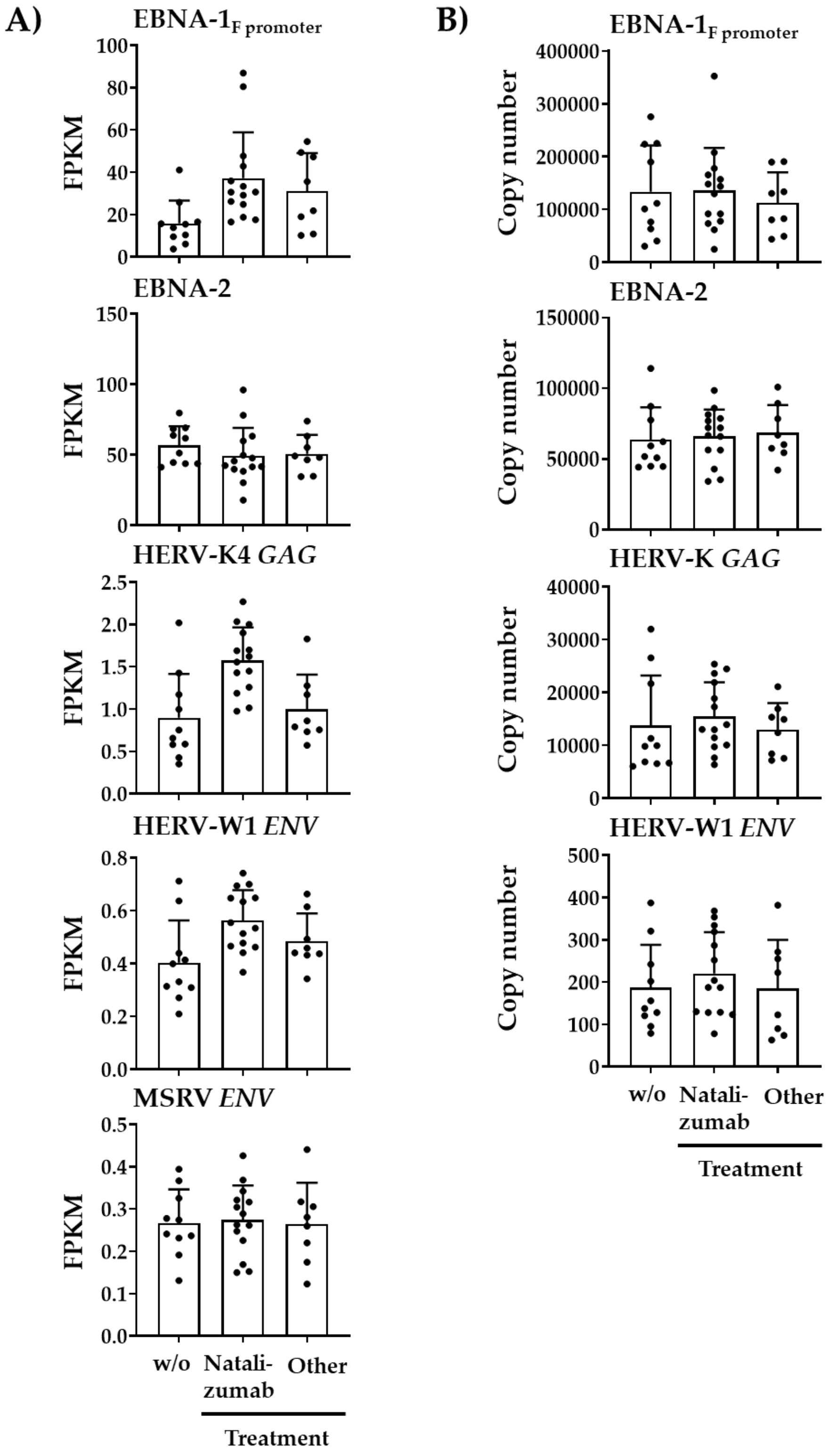

Supplementary Figure S9. Expression of EBV and HERVs in LCL from individuals with MS regarding disease-modifying therapy. The graphs show means±SD of 59 LCL from 32 individuals with MS by A) RNAseq or B) qRT-PCR. Multiple LCL from the same donor served as biological replicates. Twenty-two of all individuals with MS received therapy with natalizumab (n=14) or other drugs (n=8). These drugs include fingolimod (n=3), alemtuzumab (n=1), interferon beta-1a (n=1), carbamazepine (n=1), glatiramer acetate (n=1) and dimethyl fumarate (n=1). Ten individuals with MS were not receiving disease-modifying-therapies at the time of blood collection. Statistical significances were calculated using a two-way ANOVA with Bonferroni's posthoc test and are presented in Table 2 of the manuscript (RNAseq) or in Table S1 of the supplement (qRT-PCR).

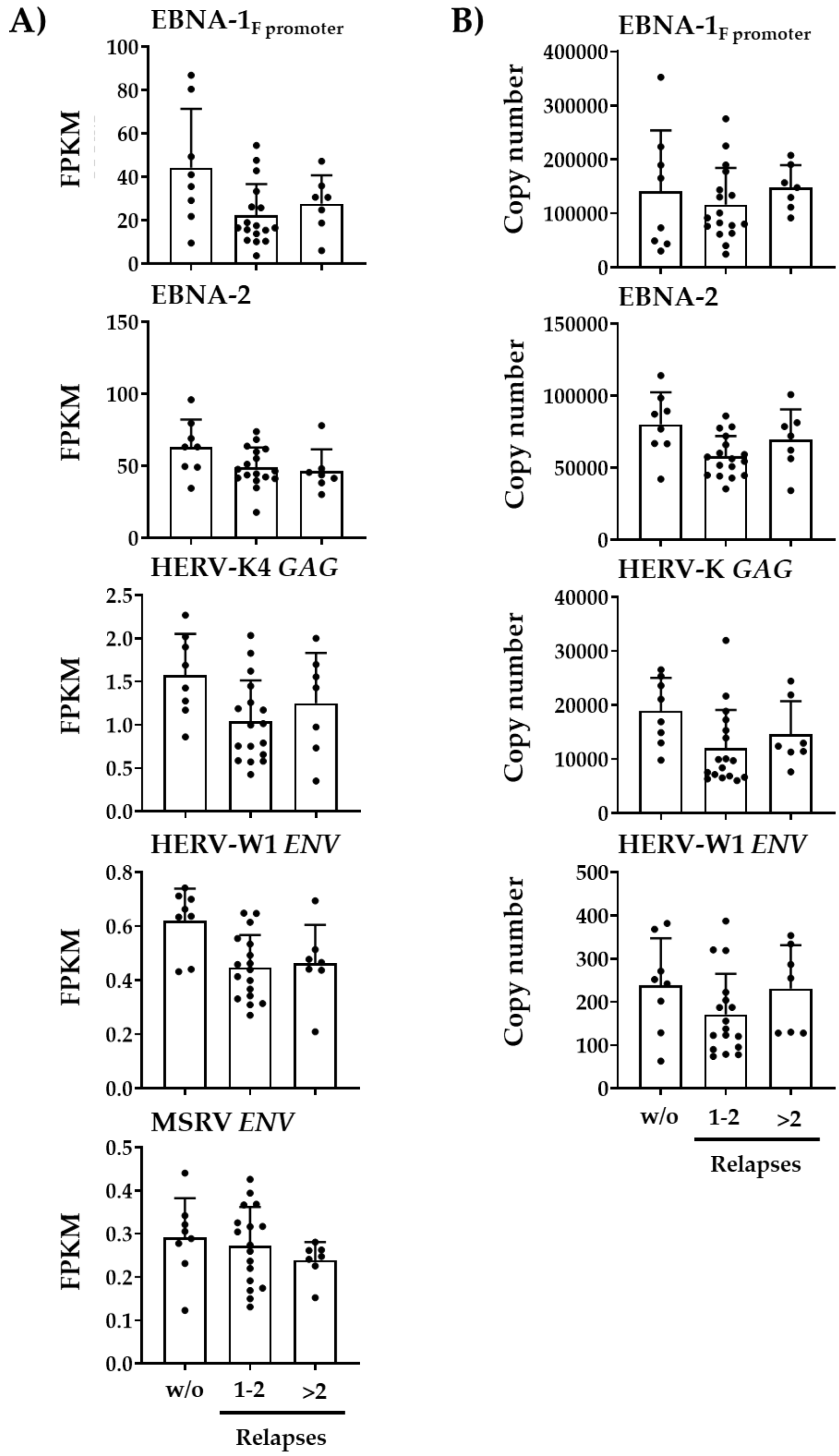

Supplementary Figure S10. Expression of EBV and HERVs in LCL from individuals with MS regarding relapse rate. The graphs show means±SD of 59 LCL from 32 individuals with MS by A) RNAseq or B) qRT-PCR. Multiple LCL from the same donor served as biological replicates. Eight of all individuals with MS were relapse-free, seventeen had one or two relapses and seven had more than two relapses within the last two years at the time of blood collection. Statistical significances were calculated using a two-way ANOVA with Bonferroni's posthoc test and indicated in Table 2 of the manuscript (RNAseq) or in Table S1 of the supplement (qRT-PCR).

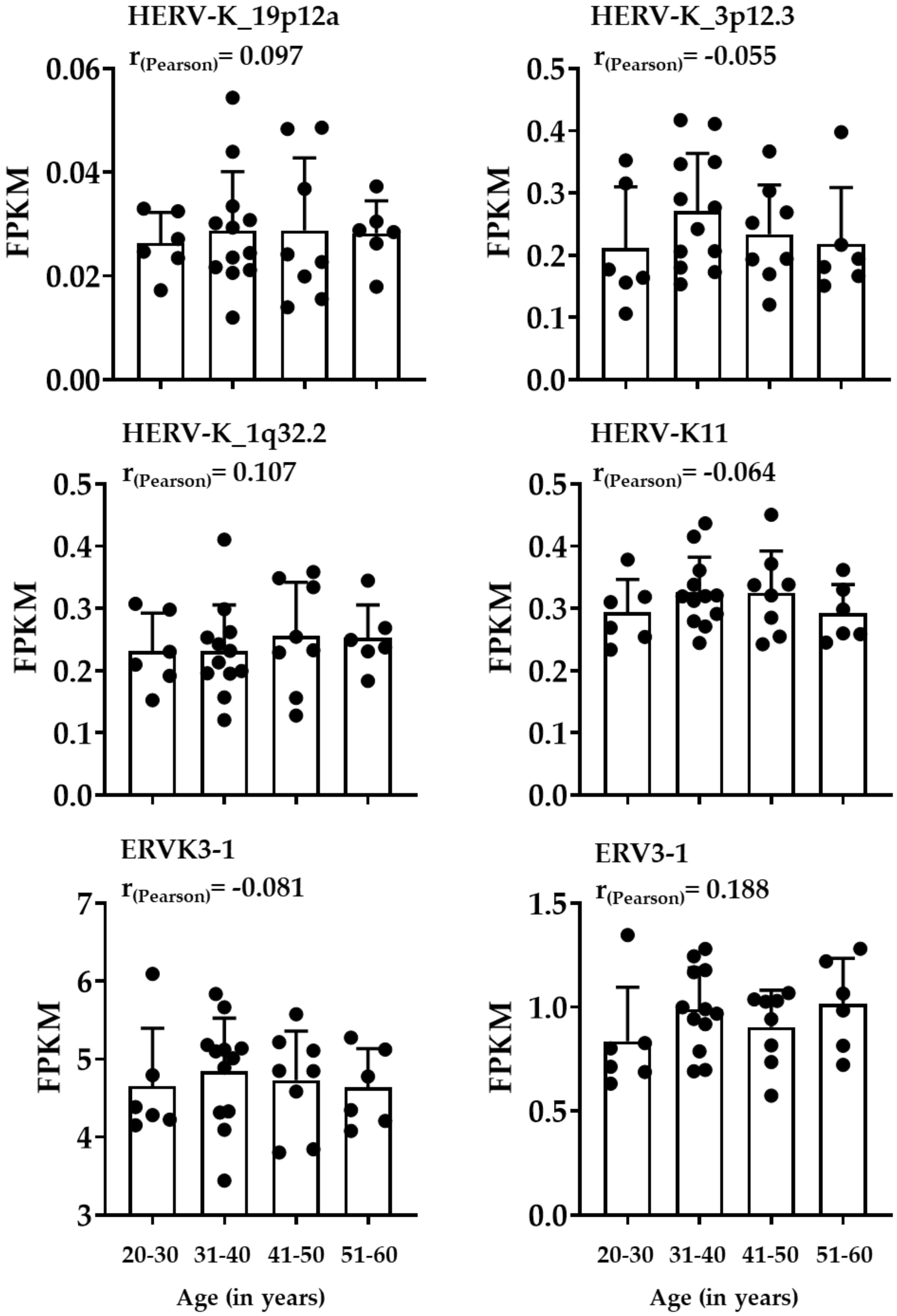

Supplementary Figure S11. Expression of HERVs up-regulated in MS regarding age of the individuals. The graphs show means±SD of 59 LCL from 32 individuals with MS analyzed by RNAseq. Multiple LCL from the same donor served as biological replicates. The correlation of HERV expression with age was indicated by Pearson's correlation coefficient. The increase of these HERV loci in MS compared to controls is presented in Figure 3 of the manuscript.

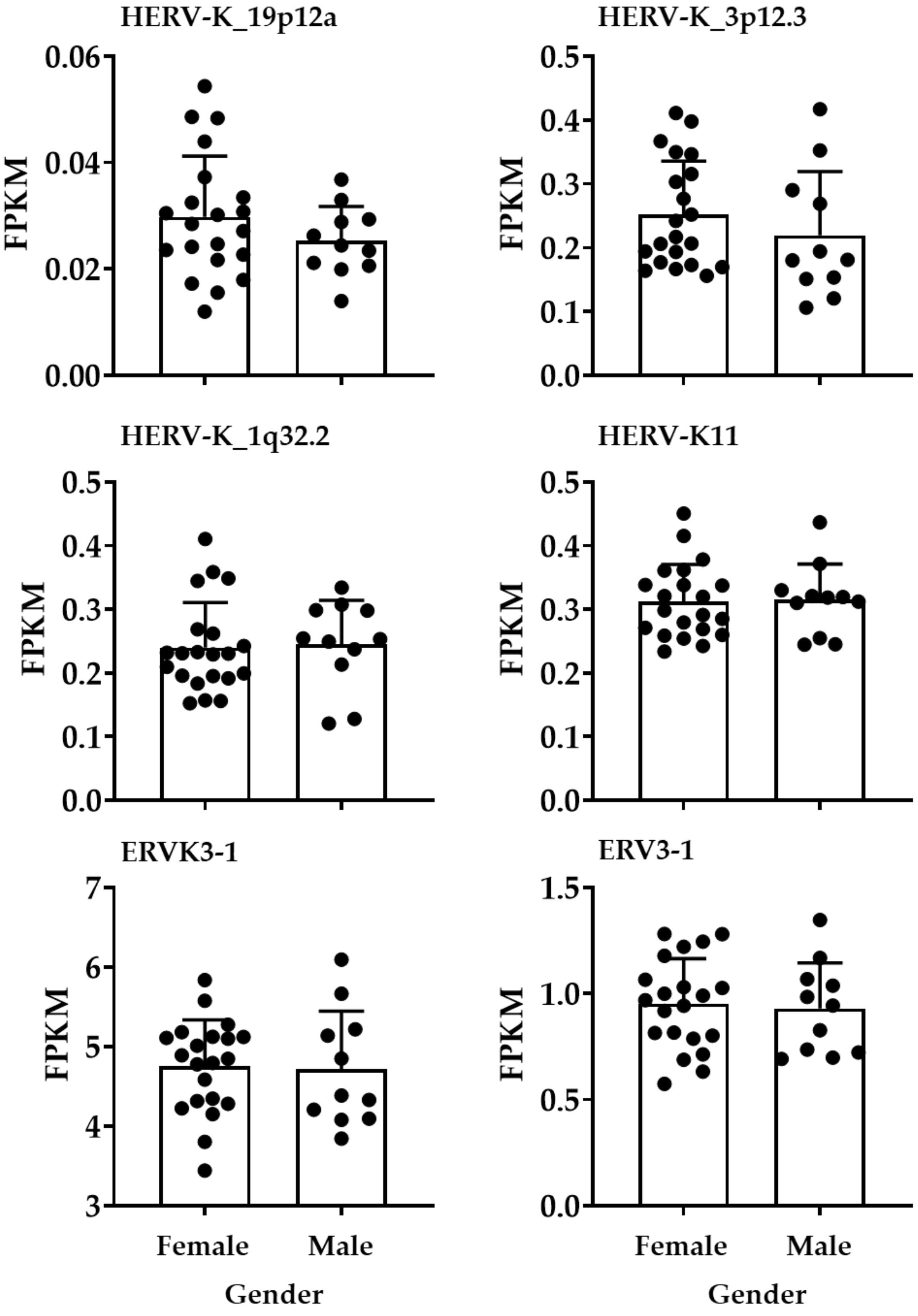

Supplementary Figure S12. Expression of HERVs up-regulated in MS regarding gender of the individuals. The graphs show means±SD of 59 LCL from 32 individuals with MS analyzed by RNAseq. Multiple LCL from the same donor served as biological replicates. The increase of these HERV loci in MS compared to controls is presented in Figure 3 of the manuscript.

Supplementary Tables

Supplementary Table S1. Influence of environmental and clinicopathological factors on EBV and HERV expression in LCL from MS individuals. Copies from the indicated transcripts were determined in 94 LCL from 32 individuals with MS by qRT-PCR. LCL from the same individual served as biological replicates. Data are means±SD. The RNAseq analysis from a part of the same LCL is shown in Table 2 of the manuscript. Statistics: Two-way ANOVA with Bonferroni's posthoc test; p values < 0.05 were indicated.

	EBNA-2	EBNA-1 F promoter	HERV-K *GAG*	HERV-W1 *ENV*
GENDER				
**Female (n=21)**	70101±21531	131151±62245	15438±7189	213.7±98.32
**Male (n=11)**	58031±13684	125605±101008	12142±6668	176.0±107.7
SMOKING BEHAVIOR				
**Smoker (n=11)**	66940±12583	145965±91374	15308±6932	241.0±113.6
**Non-Smoker (n=21)**	65435±23017	120486±67660	13780±7278	179.6±90.39
VITAMIN D DEFICIENCY				
**Deficient (n=14)**	65782±24946	125928±66590	14874±8025	187.2±83.19
**Non-Deficient (n=7)**	71167±20332	102789±64349	15401±6136	244.8±81.62
**Unknown (n=11)**	62850±11526	150302±93442	12884±6771	190.0±131.3
AGE AT STUDY ENTRY				
**Age 20-30 years (n=6)**	62030±12589	222036±89983[a]	15431±8463	248.2±133.6
**Age 31-40 years (n=12)**	69903±21808	113316±59994[b]	14402±2265	207.0±101.8
**Age 41-50 years (n=8)**	60179±24279	115278±54312[c]	13733±6161	150.4±63.96
**Age 50-60 years (n=6)**	69671±16828	86932±48996[d]	13750±6978	208.0±103.2
***P* value**		**[a] vs [c]: *P*< 0.001**<br>**[a] vs [b,d]: *P*< 0.0001**		
DURATION OF DISEASE				
**Time >10 years (n=11)**	64598±21900	108458±54903	13823±5208	166.7±97.80
**Time ≤10 years (n=21)**	66661±19195	140133±84365	14558±8005	101.1±22.07
LIFETIME WITH DISEASE[†]				
**Lifetime ≥20 % (n=15)**	68080±22778	138272±86110	15056±6546	186.9±99.91
**Lifetime ≥20 % (n=15)**	68080±22778	138272±86110	15056±6546	186.9±99.91
RELAPSERATE IN 2 YEARS				
**Relapses >2 (n=7)**	69405±21157	147896±41391	14587±6154	230.7±100.6
**Relapses 1-2 (n=17)**	57808±14217	116103±67960	12020±7094	170.7±94.51
**Relapse free (n=8)**	80238±22142	140852±112828	18917±6152	238.5±108.9
DISEASE-MODIFYING THERAPIES (DMT)				
**Natalizumab (n=14)**	65958±19054	135784±80586	15475±6442	219.9±98.11
**No DMT (n=10)**	63708±22871	133550±87457	13736±9458	186.6±101.5
**Others[‡] (n=8)**	68748±19477	112420±57850	12971±5008	184.9±115.1
EXPANDED DISABILITY STATUS SCALE (EDSS)				
**EDSS 1.0-1.5 (n=6)**	64092±21320	161319±104042	19190±9539	200.6±130.6
**EDSS 2.0-2.5 (n=11)**	73013±21177	162466±76976	14518±7374	221.0±114.1
**EDSS 3.0-3.5 (n=6)**	64328±21781	96535±48275	12004±4131	174.1±83.88
**EDSS 4.0-5.5 (n=8)**	61494±16376	95184±42677	13112±5570	198.6±91.19

[†] Calculated from duration of disease divided by age at study entry
[‡] Interferon beta-1a, carbamazepine, alemtuzumab, glatiramer acetate, dimethyl fumarate, fingolimod (n=3)

Supplementary Table S2. Strongest up-regulated genes in LCL. The expression in 79 LCL and 16 PBMC was compared by RNAseq analysis. Genes were filtered for $p < 0.0001$ from Student's t-test, fold change > 100 and ranked from highest to lowest fold change in LCL. Means±SD of EBI3 and CD70 in LCL and PBMC were shown in Figure 2 of the manuscript.

Name	Gene ID	Gene length	Fold change	*P* value
**RPS18P12**	ENSG00000230897	459	1845.695	3.64E-17
**AL080243.2**	ENSG00000237214	582	1521.000	8.27E-19
**AL355032.1**	ENSG00000241494	438	1325.957	4.92E-17
**TUBBP2**	ENSG00000214222	411	1203.464	3.28E-18
**TUBBP1**	ENSG00000127589	1646	963.355	2.66E-19
**COX20P2**	ENSG00000235013	358	735.251	6.25E-21
**C6orf223**	ENSG00000181577	3917	476.062	2.21E-34
**RPL35P1**	ENSG00000237991	370	464.138	2.42E-26
**RF00154**	ENSG00000222937	70	457.162	1.63E-24
**AICDA**	ENSG00000111732	3115	419.816	4.84E-18
**CCL22**	ENSG00000102962	2929	409.837	3.18E-10
**WDR83OS**	ENSG00000105583	2656	406.278	1.93E-30
**UBE2V1P2**	ENSG00000214192	440	397.900	1.52E-26
**LINC01055**	ENSG00000235366	1721	358.118	3.73E-26
**AC011495.1**	ENSG00000243829	584	339.879	6.41E-19
**PIEZO2**	ENSG00000154864	14796	317.221	8.87E-21
**TSPAN12**	ENSG00000106025	3368	278.091	5.92E-17
**PBX2P1**	ENSG00000244171	1291	266.579	4.60E-19
**LIPH**	ENSG00000163898	4122	244.040	1.76E-34
**UCHL1**	ENSG00000154277	2282	233.756	3.63E-16
**PRR18**	ENSG00000176381	3149	217.639	5.10E-17
**TBXT**	ENSG00000164458	2629	210.461	3.11E-08
**CREB3L1**	ENSG00000157613	4037	207.154	1.57E-22
**KCNN3**	ENSG00000143603	13598	205.670	7.51E-37
**AL365434.1**	ENSG00000225519	380	202.237	4.60E-20
**SCD**	ENSG00000099194	5362	190.252	3.28E-41
**EIF5AP4**	ENSG00000234743	465	187.840	1.15E-28
**DHFRP1**	ENSG00000188985	559	184.358	2.10E-22
**NAALADL2-AS2**	ENSG00000226779	1038	182.651	3.98E-07
**AC005674.1**	ENSG00000250413	459	176.468	2.64E-18
**SLC12A8**	ENSG00000221955	5586	175.928	1.95E-25
**PTP4A1**	ENSG00000112245	5996	171.767	6.16E-24
**AC010343.1**	ENSG00000240376	629	169.230	3.40E-18
**EBI3**	ENSG00000105246	1284	166.358	4.11E-27
**CMA1**	ENSG00000092009	937	159.748	1.75E-16
**RPL38P4**	ENSG00000250562	214	159.214	2.09E-28
**ASCL1**	ENSG00000139352	2472	157.791	4.66E-09
**CD70**	ENSG00000125726	1532	153.499	6.55E-37

**DSG2**	ENSG00000046604	6190	151.716	1.30E-24
**ENTPD2**	ENSG00000054179	2633	149.833	2.64E-12
**RPS20P14**	ENSG00000223803	360	149.346	2.31E-25
**CLIC1P1**	ENSG00000231313	724	148.516	1.87E-18
**CLDN14**	ENSG00000159261	2853	140.856	3.96E-27
**GCSHP5**	ENSG00000224837	522	138.391	2.72E-23
**AC116347.1**	ENSG00000238000	720	135.091	1.33E-24
**SUMO2P6**	ENSG00000249031	288	134.499	6.28E-23
**AL109918.1**	ENSG00000216775	3821	134.221	1.92E-25
**MYL12BP2**	ENSG00000227765	512	134.049	3.59E-19
**PIR**	ENSG00000087842	2512	133.566	2.92E-29
**LAMP3**	ENSG00000078081	3816	131.781	1.69E-29
**SERBP1P1**	ENSG00000213740	1160	130.691	4.42E-28
**MIR138-1**	ENSG00000207954	99	128.875	6.48E-19
**RHOV**	ENSG00000104140	1705	128.789	6.35E-20
**STC2**	ENSG00000113739	6128	121.871	5.79E-12
**NUSAP1**	ENSG00000137804	2875	119.434	3.72E-24
**HNRNPKP4**	ENSG00000243547	1386	118.948	3.04E-28
**AC016734.1**	ENSG00000228305	654	116.305	2.41E-26
**TMOD1**	ENSG00000136842	3708	114.141	2.85E-32
**BHLHE22**	ENSG00000180828	3262	109.369	4.36E-08
**LGALS14**	ENSG00000006659	1079	107.848	1.49E-15
**CCL25**	ENSG00000131142	989	102.408	2.31E-18
**MAP1B**	ENSG00000131711	12630	101.623	7.63E-22

Supplementary Table S3. Strongest up-regulated genes in MSLCL. The expression in 20 coLCL and 59 MSLCL was compared by RNAseq analysis. Genes were filtered for $p < 0.05$ from Student's t-test, fold change > 1.5 and ranked from highest to lowest fold change in MSLCL.

Name	Gene ID	Gene length	Fold change	*P* value
**AC087385.1**	ENSG00000240163	312	4.7733	0.0239
**Z99755.1**	ENSG00000238153	380	3.9157	0.0045
**LINC01934**	ENSG00000234663	14668	3.7429	0.0477
**CLCA2**	ENSG00000137975	4496	3.7421	0.0322
**SEZ6L**	ENSG00000100095	7149	3.2999	0.0069
**DLG1-AS1**	ENSG00000227375	1694	3.0632	0.0002
**MT-TQ**	ENSG00000210107	72	2.3671	0.0002
**ERVMER61-1**	ENSG00000230426	10142	2.2245	0.0001
**RF00012**	ENSG00000221044	204	2.0735	1.81E-05
**REELD1**	ENSG00000250673	2260	1.9752	1.08E-06
**ABHD17AP4**	ENSG00000229107	909	1.9349	0.0228
**PCOTH**	ENSG00000205861	1200	1.9214	0.0032
**CCDC13-AS1**	ENSG00000173811	2164	1.7574	0.0020
**AL356124.1**	ENSG00000226149	864	1.7018	0.0006
**LTC4S**	ENSG00000213316	1192	1.6733	0.0010
**RNU7-20P**	ENSG00000251712	62	1.6365	4.47E-06
**MPL**	ENSG00000117400	2212	1.6161	0.0002
**NOTCH3**	ENSG00000074181	9394	1.6144	0.0020
**MIR365A**	ENSG00000207725	110	1.5450	0.0331
**LINC01118**	ENSG00000222005	2267	1.5317	0.0004

Supplementary Table S4. Strongest up-regulated genes in coLCL. The expression in 20 coLCL and 59 MSLCL was compared by RNAseq analysis. Genes were filtered for $p < 0.05$ from Student's t-test, fold change > 1.5 and ranked from highest to lowest fold change in coLCL. The heatmap of these genes is shown in Figure 2 of the manuscript.

Name	Gene ID	Gene length	Fold change	*P* value
**RAD51**	ENSG00000051180	3046	3.2257	0.0187
**AC093155.1**	ENSG00000225568	826	2.5951	0.0053
**ARPC3P5**	ENSG00000214027	534	2.4604	0.0219
**ACTC1**	ENSG00000159251	4639	2.4297	0.0215
**MSLN**	ENSG00000102854	2560	2.3196	0.0074
**C16orf46**	ENSG00000166455	2376	2.2051	0.0128
**TNFSF11**	ENSG00000219451	428	1.9708	0.0035
**C4orf36**	ENSG00000163633	2100	1.9332	0.0103
**AL354702.1**	ENSG00000215895	1960	1.9142	0.0098
**PRSS21**	ENSG00000007038	1967	1.8912	0.0280
**BACE2**	ENSG00000182240	9619	1.8450	0.0038
**ANP32C**	ENSG00000248546	705	1.8374	0.0119
**DHX9P1**	ENSG00000228002	3076	1.8113	0.0021
**AC018738.1**	ENSG00000237039	210	1.7672	0.0038
**DTX1**	ENSG00000135144	4387	1.7585	0.0019
**HEPHL1**	ENSG00000181333	5345	1.7306	0.0019
**STEAP1B**	ENSG00000105889	2316	1.6934	0.0391
**INMT**	ENSG00000241644	3376	1.6817	0.0085
**AC093616.1**	ENSG00000234231	2070	1.6741	0.0266
**NPC1L1**	ENSG00000015520	5252	1.6429	0.0441
**BX322650.1**	ENSG00000183171	2143	1.6424	0.0067
**RN7SKP225**	ENSG00000222337	352	1.6397	0.0354
**COX7CP1**	ENSG00000235957	192	1.6042	0.0132
**YWHAEP1**	ENSG00000169418	5003	1.6004	0.0054
**RPS10P3**	ENSG00000217716	494	1.5987	0.0041
**CHIA**	ENSG00000134216	4632	1.5948	0.0305
**RPL3P2**	ENSG00000227939	1203	1.5937	0.0089
**IGHV1-69**	ENSG00000211973	412	1.5787	0.0491
**DEPP1**	ENSG00000165507	2979	1.5715	0.0001
**FAM89A**	ENSG00000182118	2394	1.5678	0.0263
**AC002056.1**	ENSG00000213683	760	1.5673	0.0111
**AL591623.1**	ENSG00000225300	1960	1.5655	0.0168
**LINC02381**	ENSG00000250742	2310	1.5613	0.0350
**ANK1**	ENSG00000029534	10797	1.5341	0.0011
**CXCR5**	ENSG00000160683	4431	1.5297	0.0171
**CTH**	ENSG00000213763	1123	1.5297	0.0006
**SMG1P1**	ENSG00000237296	7046	1.5104	0.0127
**RHOV**	ENSG00000074416	10102	1.5033	0.0003

**IGFBP6**	ENSG00000167779	1279	1.5023	0.0184

Supplementary Table S5. Strongest up-regulated viral transcripts in LCL. The expression in 79 LCL and 16 PBMC was compared by RNAseq analysis. Genes were filtered for $p < 0.05$ from Student's t-test. Fold change > 1 and ranked from highest to lowest fold change in LCL.

Name	Exemplary accession number	Gene length	Fold change	*P* value
**EBNA-1**	NC_007605	1926	1692.747	4.00E-44
**HERV-K18**	JN675013.1	9232	2.132	1.35E-20
**1q21.3_HERV-K**	JN675012.1	3091	1.849	3.98E-09
**12q24.11_HERV-K**	JN675069.1	1485	1.564	0.0002
**HERV-KC4**	X80240.1	6369	1.332	0.0038
**HERV-9**	X57147.1	3918	1.119	0.0393
**HERV-W5**	AC117456.6: 51035-52536	1502	1.075	0.0008
**22q11.23_HERV-K**	JN675088.1	8859	1.038	0.0030

Supplementary Table S6. Strongest up-regulated viral transcripts in MS-derived LCL. The expression in 59 MSLCL and 20 coLCL was compared by RNAseq analysis. Genes were filtered for $p < 0.05$ value from Student's t-test, fold change > 1 and ranked from highest to lowest fold change in MSLCL. The heatmap of these genes is shown in Figure 3 of the manuscript.

Name	Exemplary accession number	Gene length	Fold change	*P* value
**19p12a_HERV-K**	JN675076.1	9382	1.1989	0.0008
**ERV3-1**	NC_000007.14:64990356-65006687	16332	1.1046	0.0008
**ERVK3-1**	NC_000019: 58305374-58315657	10284	1.0352	0.0015
**HERV-K11**	JN675025.1	9180	1.0285	0.0038
**3p12.3_HERV-K**	JN675019.1	8686	1.0070	0.0024
**1q32.2_HERV-K**	JN675016.1	4213	1.0045	0.0054

Supplementary Table S7. Variability of gene expression in LCL replicates from the same PBMC donor. The sum of genes above the cut-off values listed in Table 3 of the manuscript was calculated. The sum of genes from (a) all 35 genes or (b) 15 genes proposed from them for a biomarker panel is presented for all MS individuals. Individuals without a second LCL replicate were stated as not available (n.a.).

**Donor**	**(a) Sum of genes above *cut-off* from 35 genes**		**(b) Sum of genes above *cut-off* from 15 genes**	
	**LCL replicate 1**	**LCL replicate 2**	**LCL replicate 1**	**LCL replicate 2**
**MS-001**	9	15	2	5
**MS-002**	6	3	0	2
**MS-003**	3	2	1	1
**MS-006**	8	4	2	1
**MS-008**	19	7	6	1
**MS-009**	7	n.a.	4	n.a.
**MS-010**	4	10	1	5
**MS-011**	13	n.a.	6	n.a.
**MS-012**	16	21	8	11
**MS-013**	14	15	5	4
**MS-014**	9	16	4	9
**MS-015**	3	8	1	2
**MS-016**	19	18	9	10
**MS-017**	4	n.a.	2	n.a.
**MS-018**	13	25	6	10
**MS-019**	11	16	6	6
**MS-020**	22	16	11	9
**MS-021**	11	16	5	5
**MS-022**	16	22	7	8
**MS-023**	26	17	11	8
**MS-026**	11	19	2	7
**MS-027**	5	5	4	1
**MS-028**	11	12	6	4
**MS-029**	9	n.a.	4	n.a.
**MS-030**	8	0	3	0
**MS-031**	4	6	1	1
**MS-032**	13	3	8	3
**MS-033**	5	n.a.	2	n.a.
**MS-038**	8	15	5	8
**MS-039**	22	15	10	6
**MS-040**	7	16	3	8
**MS-041**	13	n.a.	7	n.a.
